# Statistics and simulation of growth of single bacterial cells: illustrations with *B. subtilis* and *E. coli*

**DOI:** 10.1038/s41598-017-15895-4

**Published:** 2017-11-23

**Authors:** Johan H. van Heerden, Hermannus Kempe, Anne Doerr, Timo Maarleveld, Niclas Nordholt, Frank J. Bruggeman

**Affiliations:** 1 Systems Bioinformatics, VU University, De Boelelaan 1087, 1081 HV Amsterdam, The Netherlands; 2 Swammerdam Institute for Life Sciences, University of Amsterdam, Science Park 904, 1098 XH Amsterdam, The Netherlands; 3 Present Address: Department of Bionanoscience, Kavli Institute of Nanoscience, TU Delft, Delft, The Netherlands; 4 Present Address: Central Risk Management, ABN AMRO NV, Amsterdam, The Netherlands; 5 Present Address: Federal Institute for Materials Research and Testing (Department of Materials and Environment, Division Biodeterioration and Reference Organisms), D-12205, Berlin, Germany

## Abstract

The inherent stochasticity of molecular reactions prevents us from predicting the exact state of single-cells in a population. However, when a population grows at steady-state, the probability to observe a cell with particular combinations of properties is fixed. Here we validate and exploit existing theory on the statistics of single-cell growth in order to predict the probability of phenotypic characteristics such as cell-cycle times, volumes, accuracy of division and cell-age distributions, using real-time imaging data for *Bacillus subtilis* and *Escherichia coli*. Our results show that single-cell growth-statistics can accurately be predicted from a few basic measurements. These equations relate different phenotypic characteristics, and can therefore be used in consistency tests of experimental single-cell growth data and prediction of single-cell statistics. We also exploit these statistical relations in the development of a fast stochastic-simulation algorithm of single-cell growth and protein expression. This algorithm greatly reduces computational burden, by recovering the statistics of growing cell-populations from the simulation of only one of its lineages. Our approach is validated by comparison of simulations and experimental data. This work illustrates a methodology for the prediction, analysis and tests of consistency of single-cell growth and protein expression data from a few basic statistical principles.

## Introduction

Thousands of biochemical reactions are required for bacterial growth and division. Some of them operate in a regime where they are susceptible to stochastic fluctuations in the concentrations of their reactants and regulators^1,2^. These fluctuations can be amplified by molecular networks^3^, in particular by those with positive feedback circuitry and propagated to the cellular level, causing variations in the growth characteristics^4^ (birth size, division size, generation time etc.) and molecular content^5^ of individual cells. A reverberating coupling exists between the molecular composition of a cell and its growth behaviour^4^, where fluctuations at a cellular level can in turn cause cell-to-cell variations at the level of molecules and reaction activities. For instance, uneven cell division causes size differences between cells such that their protein content and reaction rates vary^4,6,7^. The fluctuating copy number of a particular molecule in a cell, over a time period of several bacterial cell cycles, is therefore the outcome of (stochastic) biochemical and cell-growth processes^8–10^.

Coupled molecular and cellular fluctuations are associated with many surprising phenomena in single-cell biology^10–14^. This complex feedback circuitry generates cell-to-cell variability in a population of isogenic cells, which may result in individual cells transiting to different phenotypic states when conditions change^11–13,15,16^. Examples include adaptive phenotypic-diversification of populations of cells, e.g. the emergence of antibiotics-tolerant persister cells^2^ and bacterial-cell differentiation^16^. Acquiring a predictive understanding of these phenomena is one of the current challenges in single-cell physiology^17^, with direct applications in biotechnology^18^ and medical microbiology^19,20^. Disentangling causes and effects, using a stochastic framework, is a major challenge for single-cell physiology^21,22^ and methods need to be developed that can quantify the contributions made by the stochastic biochemistry of molecular circuits and cellular growth, including theory (e.g. variance decomposition) and simulation^6,21–23^.

In contrast to the complexity of molecular and cellular processes at a single-cell level, the macroscopic, population-level properties of bacterial cultures are much easier to quantify and predict. In fact, the properties of bacterial cultures at balanced growth follow surprisingly simple ‘laws’. Examples are the relations of Malloe-Schaechter-Kjeldgaard^24^, Monod^25^ and Pirt^26^, which were developed in the 1950-70 s. In that same period, a statistical theory was derived for the behaviour of single cells at balanced growth – a ‘microscopic’ growth theory^27–29^. It describes statistical relations between growth properties of single cells, such as birth and division volumes, generation times, growth rates, and daughter-mother volume ratios. Importantly, these descriptions assume that the distributions of these properties are time-invariant, i.e. the probabilities to observe cells with particular growth properties are fixed, and therefore, only hold if balanced growth conditions are met. With this theory, quantitative descriptions of populations can account for inter-individual variations in the physiological parameters (i.e. their distributions) of asynchronously growing cells.

In the current work, we validate this statistical theory with real-time imaging of bacterial growth and fluorescent-protein expression, using time-lapse fluorescence microscopy. We show that relations between different phenotypic characteristics can be used in consistency tests of experimental data of single-cell growth, and the prediction of single-cell statistics. We then exploit these robust correlations to develop a fast and predictive stochastic simulation algorithm of single-cell growth and protein expression. Together, the statistical relations and the stochastic simulation algorithm offer a methodology for prediction, interpretation and tests of consistency of experimental data of the stochasticity of single-cell growth and molecular circuit dynamics.

## Results

### Describing the growth characteristics of single cells at balanced growth

In the following sections, we will validate the statistical relations captured by microscopic growth theory^27,29–31^ and show how this framework allows for a comprehensive quantitative description of single-cell growth characteristics from a limited set of physiological single-cell growth-measures. The relations we will validate make use of several concepts, which we will first explain.

**Figure 1:**
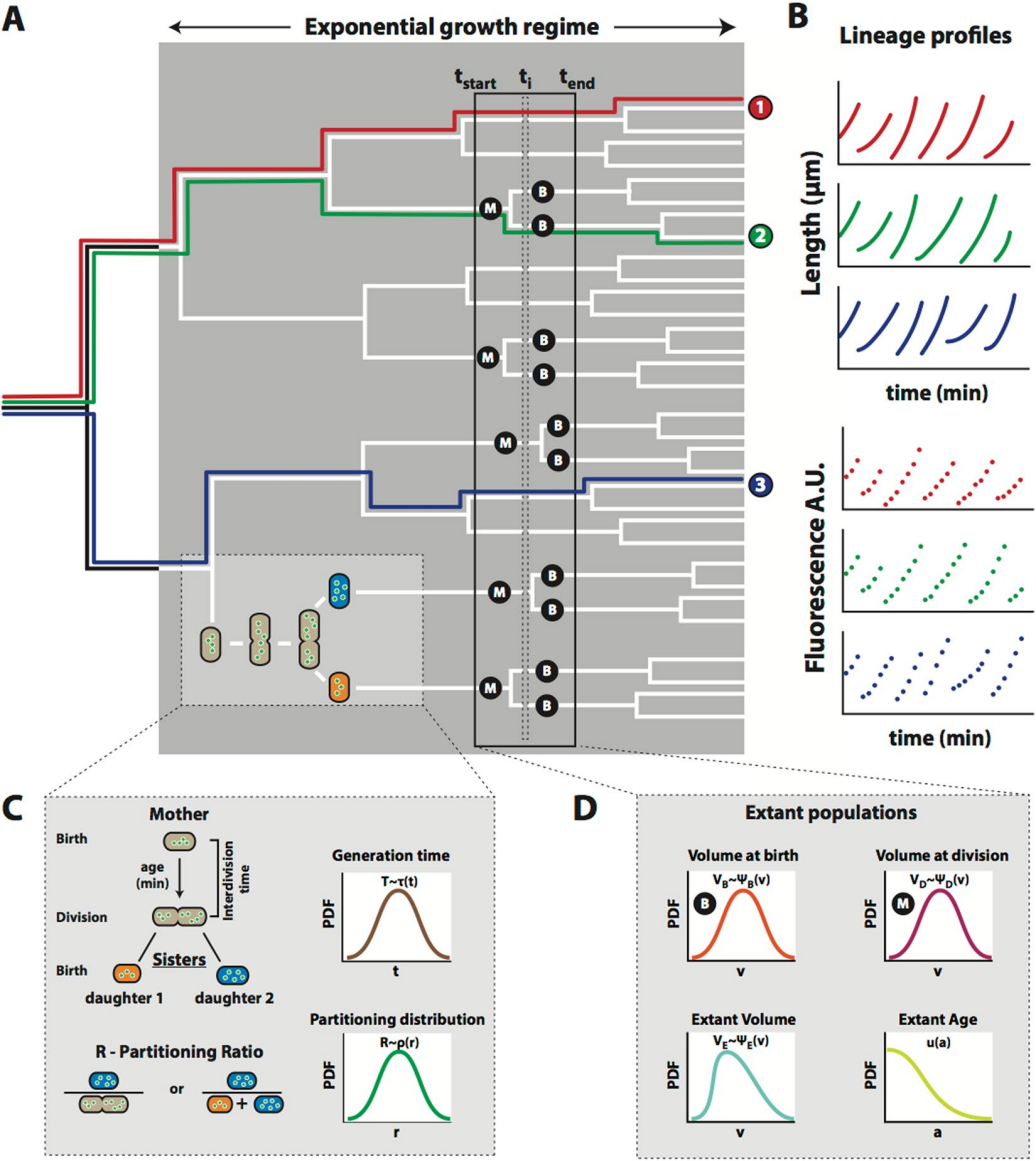
Growth characteristics and concepts of single cells in a population at balanced growth. (**A**) The formation of a microcolony from a single ancestral cell can be represented as a lineage tree. In such a tree, time runs from left to right, horizontal lines represent the life lines of single cells, their total length equals the generation time of a cell, and vertical lines indicate cell divisions. (**B**) A lineage corresponds to the growth and division of single cells, that are all daughters from a specific ancestral cell. At specific time points along a lineage, the cell length and fluorescence can be measured. (**C**) After a cell-cycle duration, corresponding to the generation time of a (mother) cell, two daughter cells arise via imperfect cell division, giving rise to a probability to observe daughter cells that have obtained a certain fraction of their mother cell’s volume and molecular content. (**D**) At one given moment in time all extant cells have particular properties that follow probability distributions such as their birth volume, division volume, current volume and current age. Extant populations consist of cells that divide (mothers, M) and cells that are born (Babies, B).

For any single cell, its age can be defined as the time elapsed since its birth. At birth, the ‘cell age’ is zero and all cellular properties are birth properties, e.g. birth-volumes, -lengths, -widths. Every cell has as its maximal age its ‘generation time’ (the generation time is also sometimes referred to as the interdivision or cell-cycle time), which is the time at which it divides and has attained its ‘division size’. The specific rate with which this volume increases, *d*ln*V*/*dt* is called the ‘instantaneous specific growth rate’. When the division volume is reached, a cell divides and partitions its volume and molecular content to yield two new cells. The dividing cell is referred to as the mother cell and the two newly formed cells as the daughters, which are sisters. The size and molecular content of each of the two daughters can be expressed as a fraction of their mother’s to capture cell-division variation. All these properties together (cell age, generation time, birth and division sizes, daughter-to-mother ratio’s and instantaneous growth rate), capture the essential information needed for a microscopic theory of growth^27,29–31^ (see Fig. 1 and the appendix for additional information related to concepts of single cell growth).

In any population these single-cell growth-measures will show variation from cell to cell, necessitating a statistical framework to understand how population-level characteristics relate to single-cell phenotypes, and how different single-cell growth-measures depend on each other. Answers to these types of questions are greatly simplified when populations are studied under conditions of balanced growth. A defining characteristic of balanced growth is that the probabilities to observe cells with particular growth properties – their phenotype – are fixed and the associated probability distributions are therefore time invariant (See also Fig. S4). Importantly, the validity of the statistical relations captured by the microscopic growth theory rests strongly on the assumption that the population being described is at balanced growth.

**Figure 2:**
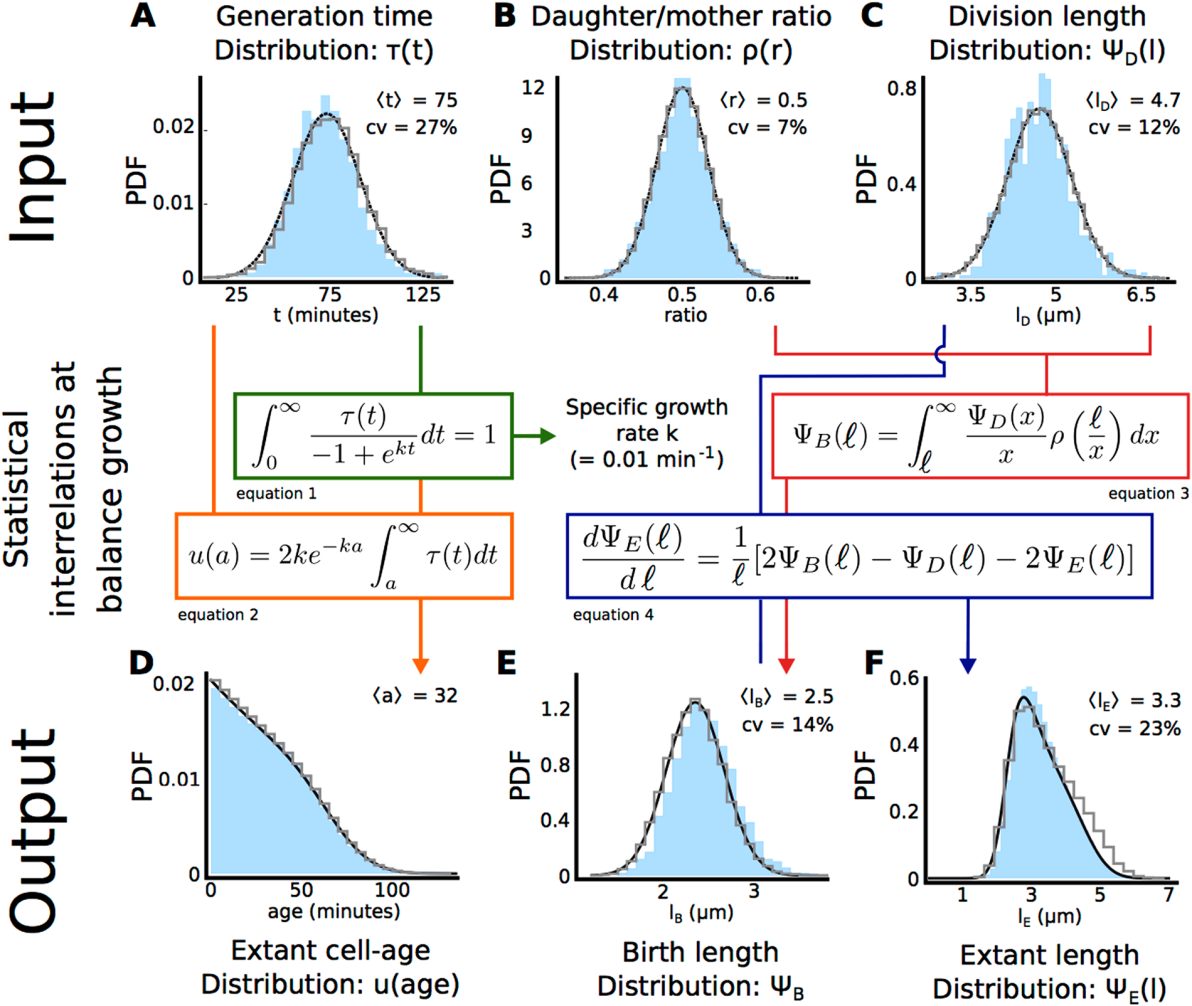
Validation of relations between growth characteristics at balanced growth for *B. subtilis*.Shown are results comparing the microscopic growth theory relations derived by Collins & Richmond^29^, Powell^28^ and Painter & Marr^27^ and experimental single-cell growth data. In this figure we validate those relations. The probability distributions obtained from experimental data are shown in blue, the validated theoretical relations are shown in the coloured boxes (eqs 1–4), the predicted distributions are shown in black in (**D**), (**E**) and (**F**), and the results of our simulation algorithm (discussed in the final result section) are shown as grey histograms. In (**A**–**C**), black lines indicate fits. We calculated the population specific growth rate from the distribution of the generation times (**A**). The distribution of the cell ages (**D**; age is the time elapsed since birth) can be obtained from the generation time distribution (**A**) and the calculated specific growth rate of the population. Using the distribution of the division lengths (**C**) and the birth lengths (**E**), the distribution of cell length of all cells that exist at a particular moment in time can be obtained. The distribution of birth lengths (**E**) can, in turn, be obtained from the distribution of daughter-mother-volume ratios (**B**) and distribution of division lengths (**C**). Sample sizes: The sample sizes for the experimental data are 3637 extant cells, 2726 cells at birth and 1466 cells at division. Data for *E. coli* can be found in Fig. S5.

Balanced growth, being a stationary process, has as a requirement that the specific growth rate of the population remains fixed over a time period that is several times longer than the mean generation time. As such, the single cell growth data we use to validate the microscopic growth theory^27,29–31^ was confirmed to meet this requirement. By individually tracking the growth of *B. subtilis* and *E. coli* cells on agar pads, we quantified the specific growth rate of the population from the increase in the total cell length of all monitored cells, and selected data from the time-window during which the growth rate remains fixed. We confirmed that the balanced-growth period lasted for several generations and that the probability distributions of growth measures are constant during in this window^27^ (see Fig. S4). All growth measurements of *B. subtilis* can be found in Fig. 2 (discussed below) and those of *E. coli* are shown in the Supplemental Information (Fig. S5).

### The population growth rate calculated from single-cell generation times

The first statistical relation we validated allows for the calculation of the population growth rate (*k*) from the distribution of the generation times (Fig. 2, equation 1)^27,30,31^. In the macroscopic theory of bacterial growth of cell populations, the specific growth rate equals *k* = ln2/*τ*_*g*_, with *τ*_*g*_ as the generation time (also called the doubling time). Due to inter-individual variations in generation times, the macroscopic relation is inexact and the relation ${\tau }_{g}=\frac{\mathrm{ln}\,2}{k}+\frac{k{\sigma }_{t}^{2}}{2}$ has been proposed as an improved approximation, with ${\sigma }_{t}^{2}$ as the variance of the distribution of generation times^27^. The equation we used (derived in^27^; equation 1 in Fig. 2) obtains the exact value of the growth rate from the distribution of generation times.

We calculate from Painter & Marr’s relation a growth rate of 0.61*hr* ^−1^. When we compare this growth rate to the one we measured from the length increase of the population data (Fig. S2A), we find a difference of only 2.7%. From the measured growth rate, we can calculate the generation time, from ln2/*k*, which gives 70 *min*. If we use the approximate relation to calculate the generation time from the growth rate (0.01 *min* ^−1^) and the measured generation-time variance of 388 *min* ^2^, we find a generation time of 71 *min*, indicating that the approximate relation shows an error of 1.4%. We conclude that the measured probability distribution of the generation times indeed allows for a calculated value of the cell-population’s growth rate that is in agreement with an independent direct observation of the growth rate from the increase in length of the entire population of cells.

The generation-time distribution, *τ*(*t*), that we measured (Fig. 2A) can be accurately approximated by a normal distribution, which has also been observed by others^5,32–35^. The coefficient of variation of the generation time is 27%, which indicates that 16% of the cells have a generation time below 53 *min* and the generation time of the same percentage of cells exceeds 92 *min*. Significant variations in generation times therefore occur in steady-state growing bacterial cell populations. Other work has recently shown that the growth rate, the length increase during a cell cycle and the generation time are interrelated^5,32,36^.

### Obtaining the distribution of cell ages from the generation times of single cells

Since all cells grow asynchronously during balanced growth and because balanced growth is a stationary process, the probability that a cell is observed with a particular age – the time elapsed since its birth – is constant. The age of a cell ranges from 0 to its generation time, when it divides.

Painter & Marr^27^ showed that the distribution of the probabilities for the occurrence of a particular cell age can be obtained from the distribution of the generation times and the growth rate (Fig. 2, equation 2). Here we validate this theory by showing that a theoretically calculated age distribution, using Painter & Marr’s relation, closely matches the independently-observed cell age distribution (Fig. 2D), indicating that the theory holds for our dataset. This finding also confirms that, during the time window of data sampling, the cells were indeed growing balanced.

We observed that the mean age of extant *B. subtilis* cells equals 32 *min* (Fig. 2D). Since the mean generation time (the mean division age) equals 75 *min*, the average cell has a cell cycle progression of 43%. The cell-age distribution also indicates that a cell population in balanced growth contains more young than old cells, which is expected since a mother cell always divides into two daughter cells.

### The distribution of daughter-over-mother cell sizes calculated from the distributions of birth and division sizes

Powell^28^ derived a relation between the distributions of birth and division volumes and the distribution of daughter-over-mother volume ratios (Fig. 2, equation 3). This theory is in terms of the volume distributions of cells. Here we apply it to analyse cell length data (Fig. 2C). Throughout this paper, we treat length changes proportional to volume changes because the width of *B. subtilis* and *E. coli* cells remains roughly constant during growth (Fig. S3 and^5,37^). Therefore, the growth in cell volume is proportional to the growth in cell length.

We approximate the daughter-over-mother length ratio in our experimental data using the ratio of one daughter cell over the sum of her length and that of her sister (Fig. 2B). Since we monitor growth at 1-minute intervals, during which some growth takes place, a small discrepancy occurs between our estimate of the daughter-vs-mother length ratio and the real value (see also supplement information section 5).

For symmetrically dividing bacteria, such as *B. subtilis* and *E.coli*, the average daughter-over-mother length ratio is expected to be 0.5, which is reflected by our data (Fig. 2B). Application of the Powell relation (equation 3, Fig. 2) leads to a prediction of the birth length distribution, that closely matches the experimental data (Fig. 2F).

The distributions of division length, birth length and daughter-over-mother length ratio’s that we measured can all be accurately approximated by normal distributions. The daughter-over-mother length ratio has the expected mean of 0.5 and a coefficient of variation of 7%, which captures division noise. The variation in division and birth length is about twofold greater. The birth and division lengths of cells also correlate with each other (Figs S7 and S8), which is in agreement with an adder-like size-homoeostasis mechanism described for both *B. subtilis* and *E. coli* ^5^. Our data, however, deviates slightly from perfect adder behaviour, which could be due to the use of agar pads, where the formation of micro-colonies could lead to differences between cells in the centre and those at the periphery of the colony.

### Calculation of the length distribution of cells

Collins & Richmond^29^ published a relation between the growth rate as a function of cell volume and the probability distributions of daughter, mother and extant cell-volumes. When the cellular growth rate is fixed as a function of the cell volume, as it is for our data (see Fig. S10), the Collins-Richmond relation simplifies to equation 4 of Fig. 2. We used this relation to predict the extant cell length distribution from the length distributions at birth and division. Figure 2E indicates that the predicted and measured extant cell length distributions are in close agreement. Variability in the extant cell length distribution is due to the fact that both daughter and mother cells occur in the extant cell population, and their lengths (or volumes) vary on average by a factor of two. Any additional spread in this distribution arises from noise in distributions of the birth and division length.

### Exploiting the microscopic growth theory for inference of probability distributions from data

Now that we have concluded that the microscopic growth theory is in agreement with single cell growth data, we can consider its applications. One application is its use in consistency checks of experimental data, as we did in Fig. 2; in order to test whether measurements of different growth characteristics relate to each other as we theoretically expect them to. Another application of the microscopic growth theory is its use in the inference of distributions of growth characteristics of single cells when not all of them can be directly measured.

**Figure 3:**
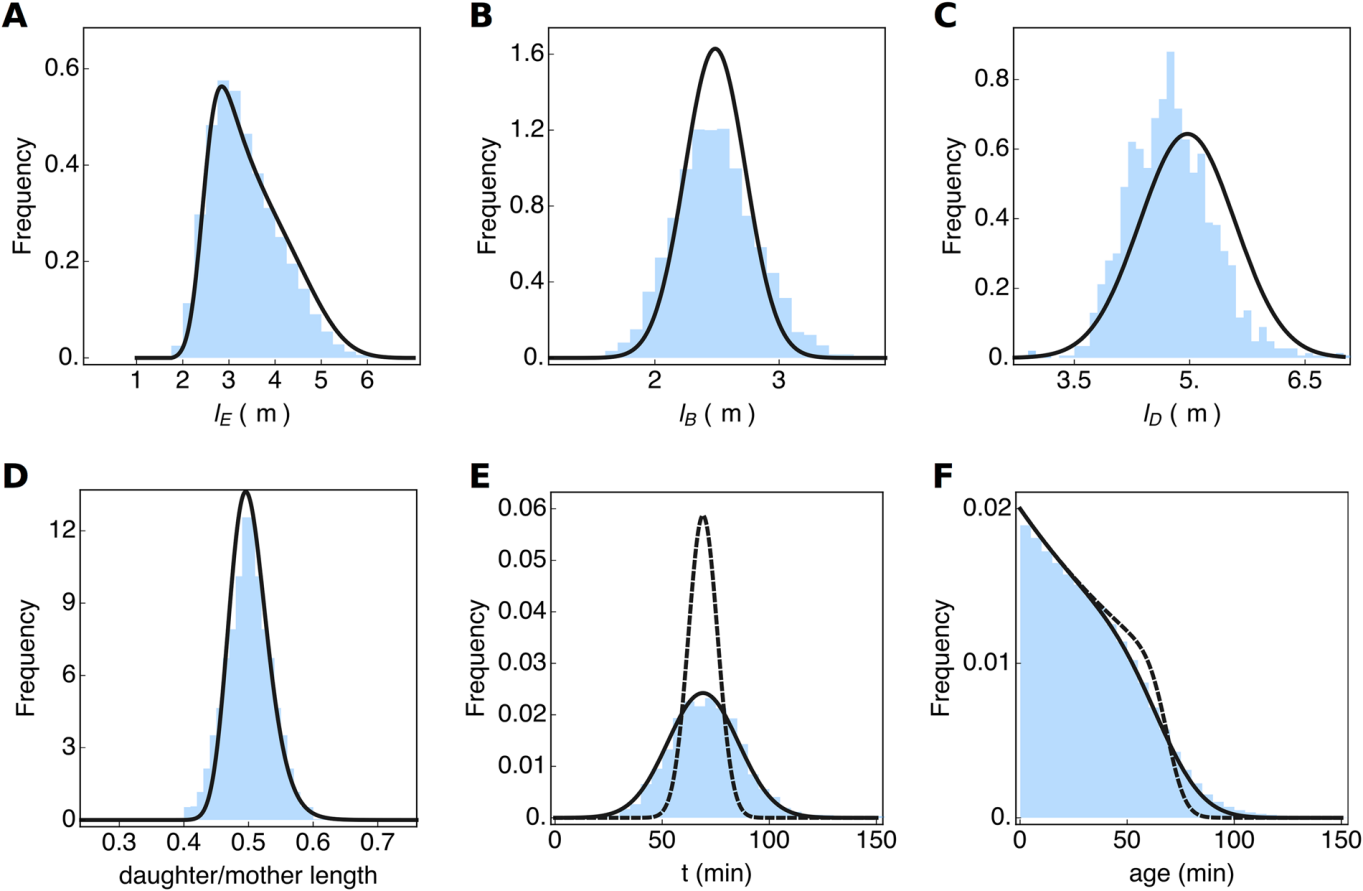
Inference of probability distributions of single-cell growth characteristics from experimental data. In this figure, the blue histograms are the measured data (also shown in Fig. 2) and the black lines are inferred from the data. (**A**) Shows the extant cell-length distribution. The cell length distributions at birth (**B**) and division (**C**) were obtained by a fit of the Richmond & Collins relation (equation 4 of Fig. 2) to the extant cell length distribution, given the measured growth rate. The distribution of the ratio of the daughter cell length over the mother cell length (**D**). It was obtained from the distributions shown in (**B**) and (**C**), assuming a correlation between cell length at birth and division of 0.85. By assuming deterministic exponential growth, the distribution of generation times (**E**), was predicted from the length ratio distribution (**D**), without noise (dashed line) and with additional noise (full line). The distribution of cell ages (**F**) was calculated from the generation time distribution (**E**), using equation 2 from Fig. 2. The dashed line in (**F**) corresponds to the dashed generation time distribution in (**E**).

Consider, for instance, a growth experiment carried out in a shake flask, where the specific growth rate and the probability distribution of the (extant) cell volumes, shown in Fig. 3A was measured (there many non-microscopy based methods for measuring cell size distributions, e.g. the Coulter counter). We shall assume that the birth and division length distributions each follow a normal distribution with means that differ by a factor two; an assumption that is in fact in agreement with our data (Fig. 2). This leads to three unknown parameters: two standard deviations and one mean. Those we can obtain by fitting the division and birth-length distributions to the measured extant length distribution, using the Collins & Richmond relation (equation 4 in Fig. 2), parameterised with the measured growth rate. In this way, the probability distributions for the birth and division volume can be inferred, those are the solid lines in Fig. 3B and C.

To determine the distribution of the daughter over mother lengths, *ρ*(*r*) with *r* = *l*_*b*_/*l*_*d*_, we use a mathematical relation that expresses this distribution in terms of the joint distribution of *l*_*b*_ and *l*_*d*_ values^38^. We assume this joint distribution to be a bivariate normal distribution, which is in agreement with the data shown in Fig. 3B and C. We still need to assume a correlation coefficient for *l*_*d*_ and *l*_*b*_ values, which we can do by assuming a sizer, adder or timer model of cell growth^5,39^. When we assume an adder then the correlation coefficient will be close to 1. If we take a correlation coefficient of 0.85, we obtain a perfect fit of the estimated distribution of daughter over mother lengths with the experimental data (Fig. 3D), indicating this estimation method works. The fit quality is, however, strongly dependent on the exact value of the correlation coefficient between *l*_*d*_ and *l*_*b*_.

At balanced growth, with a fixed growth rate along the cell cycle (assumption 3), the following relation holds (*l*_*d*_)/(*l*_*b*_) = *e* ^*kt*^ = 1/*r* with *k* as the growth rate and *t* as the generation time. We can determine the distribution of generation times, *t*, from the distribution of the daughter-over-mother volumes, using the ‘change of random variable method’, which leads to the relation *τ*(*t*) = *ke* ^−*kt*^ *ρ*(*e* ^−*kt*^). This allows us to determine the generation time distribution from the distribution of daughter-over-mother lengths. It is shown in Fig. 3E by the dashed line. It does not provide a good fit of the data, which is likely due to the fact that the assumed deterministic growth model *l*_*d*_ = *l*_*b*_ *e* ^*kt*^ misses a stochastic component. The addition of an additional noise component to the fitted generation time distribution indeed leads to a perfect fit of the data and the model. What this stochastic component is, is unclear at the moment, but it is very likely that the growth rate, *k*, cannot be assumed to be independent of cell size, *l*; which is in agreement with experimental data^34^.

Finally, the cell-age distribution can be determined from the estimated generation time *τ*(*t*) distribution (Fig. 3E), using equation 2 of Fig. 2. The resulting correspondence of the estimated distribution and the experimental data is shown in Fig. 3F. (The dashed line corresponds to the dashed generation time distribution in Fig. 3E.)

### Exploiting the microscopic growth theory in a stochastic simulation algorithm

Another interesting aspect of the microscopic growth theory is its potential utility in the development of a fast stochastic simulation-algorithm of single-cell growth and molecular circuit stochasticity at balanced growth. Stochastic simulations of single-cell growth are very challenging, because of a large computational burden; they have to simulate tens of thousands of cells as they progress through their cell cycle and divide (giving rise to the expanding lineage tree). To circumvent this computational bottleneck, many existing approaches simulate only a single lineage, but do not retrieve the growth statistics of the entire lineage tree, thereby limiting meaningful comparisons between simulations and experimental data.

The algorithm that we developed simulates growth and molecular stochasticity of a single specific lineage, from which the statistics of the entire lineage tree can be calculated using the microscopic growth theory. First, we will briefly outline this algorithm (details are provided in the Supplemental Information, Section 7). Following this, we validate the algorithm using the experimental data introduced above, including single-cell data of a fluorescent gene expression reporter.

The input of the simulation algorithm for single-cell growth is based on several of the distributions of growth measures, quantified (or assumed) at balanced growth (Fig. 2). We start by calculating an initial cell volume, a birth volume *v*(0), from the product of two random variables, one sampled from the division-volume distribution, Ψ_*D*_(*v*) (Fig. 2C), and another from the division-ratio distribution, *ρ*(*r*) (Fig. 2B). Next, given our data, we assume exponential growth for single cells (Fig. S2), with a constant, specific growth rate that is equal to the population growth rate (*k* in Fig. 2)). From the difference between the division and birth volume, and the specific growth rate *k*, we can calculate the generation time (using $T=\frac{1}{k}\,\mathrm{ln}\,\frac{v(T)}{v\mathrm{(0)}}$). At the generation time, *T*, the cell divides into two daughter cells. One daughter receives a volume *v*_*d*1_(0) = *rv*(*T*) (the value of *r* is drawn from the division-ratio distribution), which means that the second daughter’s volume will equal *v*_*d*2_(0) = *v*(*T*) − *v*_*d*1_(0).

Since our algorithm only simulates a single lineage, and not the whole lineage tree, we track only one of the two daughter cells, which makes the algorithm very computationally efficient. The current limitation of the algorithm is that it cannot deal with proteins that set the growth rate. Therefore, it simulates the stochasticity of biochemical circuits, while they are embedded in a cell that is growing in a stochastic manner, in line with the growth-statistics relations of balanced growth.

The algorithm is restricted by two conditions. Firstly, the division volume should be independent of any cellular characteristics, such as the volume at birth or any molecule concentrations. Secondly, the growth law of a cell should be deterministic and independent of the cell’s state; for instance, an exponential growth of volume with time, which is what is usually assumed.

The simulation algorithm simulates growth of cells that grow according to sizer and’sizer-like adder’ mechanisms, which is how *B. subtilis* and *E. coli* grow (Figs S7 and S8). The algorithm can also be used to simulate cells that grow as pure adders^5^. For adders, the volume at division is determined by the addition of a birth volume (drawn from the *V*_*B*_ distribution) and an added volume value (drawn from a Δ*V* distribution). When it is assumed that the mother cell divides perfectly to yield two equally sized daughter cells, the algorithm simulates cell growth of a perfect sizer. The ratio between the partitioning variability and the division volume variability determines the slope in the 〈Δ*l*|*l*_*b*_〉-vs-*l*_*b*_ plot, and for the algorithm it falls between −0.2 and −1 (in accordance with data Fig. S7).

To be able to derive the statistics of the entire lineage tree from a single simulated lineage, a specific daughter cell is selected. The daughter that is selected is chosen with a probability that equals the fraction of descendants it is expected to contribute to the population (see Supplemental information section 8.1). After daughter-cell selection, we start simulating the next cell cycle, by sampling a new division volume, etc. We repeat these steps until the lineage simulation has generated data for stationary distributions of growth measures. The statistics of the entire lineage tree can be obtained by exploiting the cell-age distribution as explained in the Supplemental Information.

We implemented this algorithm in StochPy^23^, which is available for download from http://stochpy.sf.net. A full description of the cell growth algorithm and how it is coupled with a stochastic simulation algorithm for biochemical networks can be found in the Supplemental Information (Section 8). Importantly, we show that the stochastic simulation algorithm is in agreement with analytically solvable models (Supplemental Information). In the next section, we compare simulation results with experimental data.

### Validating the simulation algorithm with single-cell growth and protein expression data of *B. subtilis* and *E. coli*

**Figure 4:**
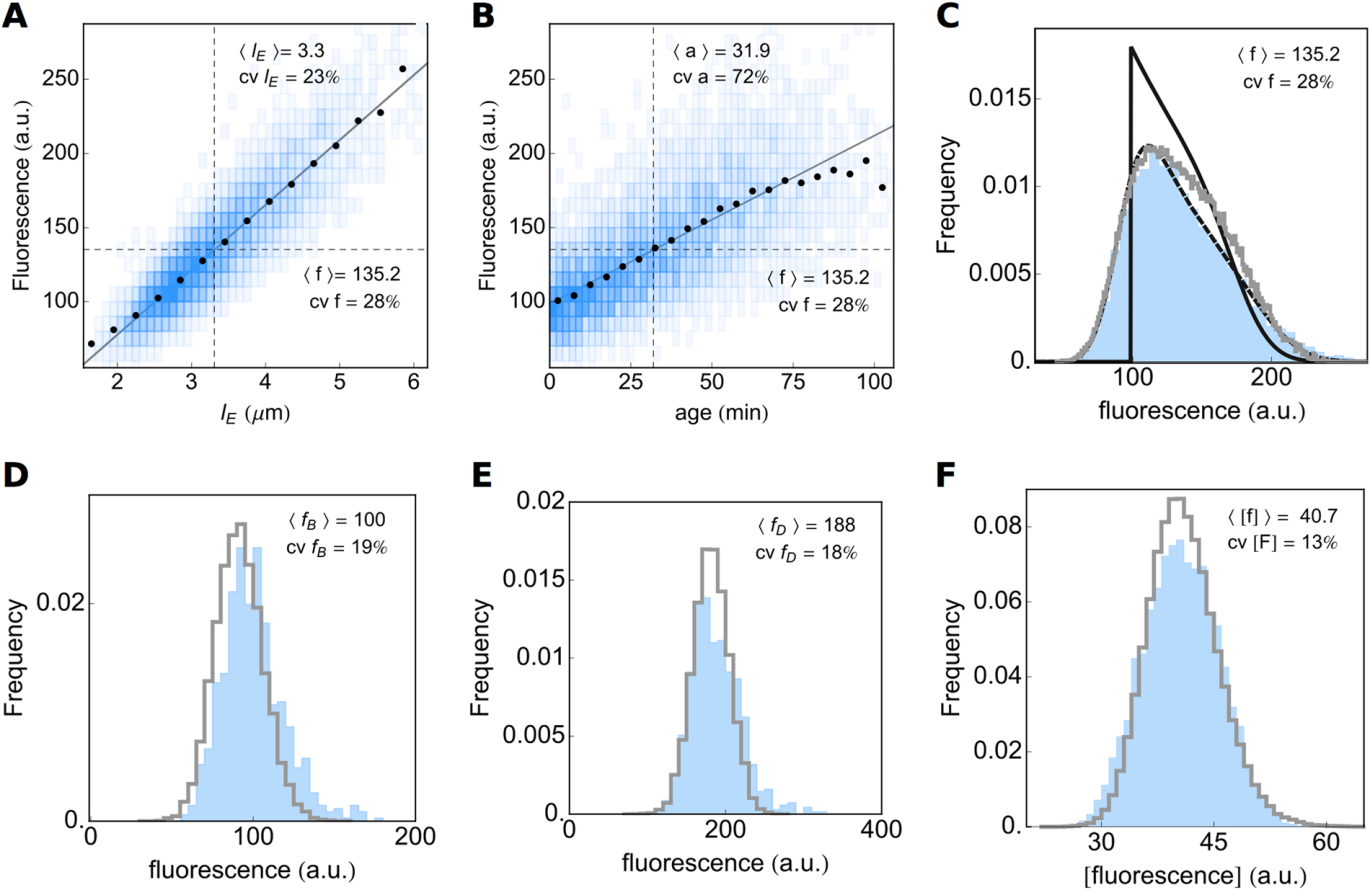
Expression data of the reporter gene of *B. subtilis*. Fluorescent protein expression scales linearly with cell length (**A**) and cell age (**B**), but the correlation is weaker for age. By transforming the extant length (Fig. 2E) and age (Fig. 2D) distributions with the linear relation between length and fluorescence and between age and fluorescence, respectively, predictions of the fluorescent distribution can be made. The result clearly shows that cell length (**C**, dashed line) is a much better predictor of measured expression levels (**C**, blue area), than age (**C**, solid line). Also shown, is the distribution of expression levels obtained by stochastic simulation (**C**, gray line). Measured fluorescence distributions at (**D**) birth and (**E**) division (blue areas) are compared to stochastic simulations (gray lines). (**F**) Shows the comparison of the measured distribution of the fluorescence concentration of all extant cells (blue) and the simulations (gray line).

The *B. subtilis* and *E. coli* strains that we used in our time-lapse microscopy experiments (Fig. 2 and Fig. S5) each expressed a genome-integrated, constitutively-expressed fluorescent protein. We used the fluorescence values of single cells as a read out of single-cell protein expression. We tested whether the stochastic simulation algorithm could reproduce single-cell growth and expression data (Fig. 4).

During balanced growth, concentrations of constitutively expressed proteins remain fixed; the cell’s content of a constitutively expressed protein therefore increases at the same rate as the cell volume. Our data clearly show that the total fluorescence levels (in arbitrary units) per cell correlate strongly with cell age (Fig. 4A and S6A) and cell length (Fig. 4B and S6B). This is to be expected, as cells that are older tend to be larger and are closer to having doubled their molecular content; the latter being a requirement for single cells under balanced growth. Although cell length and age strongly correlate, cell length explains more of the expression variability than age in both *B. subtilis* and *E. coli*, 79% and 73% vs 50% and 53% respectively. This indicates that the fluorescence signal (molecular content) of a cell is proportional to its volume (length in our case), as is expected for constitutive gene expression at balanced growth.

The relation between the mean fluorescence conditional on cell length, i.e. 〈*f*|*l*_*E*_〉, and cell length of extant cells (Fig. 4A and S6A), *l*_*E*_, can be transformed into the fluorescence distribution of the extant cell population when we take into account the cell age distribution and the dependency of *l*_*E*_ on cell age. This gives rise to a good correspondence with the measured fluorescence distribution (Fig. 4C and S6C), indicating that the gene-expression noise – independent of cell age and cell length – averages out, due to a constant expression noise during the cell cycle (Fig. S11B and D). The gene-expression noise is best captured by the noise in the fluorescence concentration (fluorescence per fixed cell area), which is shown in Figs 4F and S6F. The coefficient of variation in the fluorescence concentration equals 13% for *B. subtilis* and 14% for *E. coli*. Taken together, the fluorescence data indicate that more than 50% of the noise in total cell fluorescence originates from volume variability.

To test whether our algorithm accurately predicts cell growth and fluorescence distributions of populations at balanced growth, we performed stochastic simulations (see the Supplemental Information for details). As input we used the division length distribution (Ψ_*D*_(*l*), Fig. 2C), the daughter-over-mother volume ratio (*ρ*(*r*), Fig. 2B), and the measured growth rate (*k*). The expression model only accounts for constitutive zero-order synthesis of protein and protein dilution into new cells (no degradation). The parameters of the stochastic model of rate constant of protein synthesis was obtained by linear fitting of the mean of the logarithm of fluorescence as function of time (from Fig. 4B). To relate the measured protein fluorescence value to the protein copy number per cell used in the simulation, we use an arbitrary conversion constant. All stochastic simulation parameters can be found in the StochPy scripts available as supplemental material.

The correspondence between simulated and measured distributions, shown in Figs 2, 4, S5 and S6, indicates that the algorithm accurately recovers the statistical relations inherent in our experimental population.

It is interesting to note that the generation time distribution is used to calculate the average growth rate, however, its variability is not used as an input in the simulation algorithm. This implies that the variability observed in the generation time can be fully explained by the variability in the added volume per generation, Δ*L*. The overestimation in the fraction of large cells in the extant population (Fig. 2E) is propagated to the fluorescence distribution (Fig. 4C). Additionally, we observe a slight offset in the fluorescence distributions at cell birth and division (Fig. 4D,E). This shift is expected since our experimental setup recorded fluorescence with a resolution of ten minutes (whereas length is recorded every minute), leading to a shift between simulated and measured distributions. Fluorescence values are acquired, on average, 5 minutes after birth or 5 minutes prior to division, respectively. However, when the fluorescence concentration (Fig. 4E) for the experiment and the simulation is compared, we find a near perfect match.

## Discussion

We have validated a microscopic growth theory of bacterial cell growth with experimental data, illustrated how this theory can be exploited in the inference of probability distributions of single-cell growth characteristics, and employed the theory in the development of a fast and accurate stochastic simulation algorithm of the dynamics of a molecular circuit inside a growing single cell. This paper therefore contributes a quantitative methodology to single-cell physiology, a field that is in rapid development.

The microscopic theory relates the probability distributions of growth characteristics of single cells, such as cell sizes at birth and division, the generation time of cells, the growth rate of the cell population, the distribution of cell ages and sizes in the entire population of cells. Developed during the 1950-70 s^27–29^, the theory describes the stochasticity of the growth of symmetrically dividing single cells, under the assumption that the cell population is at balanced growth. By comparison with real-time imaging of *E. coli* and *B. subtilis* growth, we show that the microscopic theory of cell growth has stood the test of time, and provides robust descriptions of the statistics of single-cell growth.

The microscopic growth theory has several applications. For example, it provides a robust way to check the consistency of experimental data assumed to be obtained at balanced cell growth. This would involve carrying out the same procedure as outlined in Fig. 2. Essentially, predicted distributions can be compared with measured ones. In this way, one not only checks whether cells were growing balanced, but also whether the algorithms used for time-lapse analyses are accurate. The theory could therefore also be conceived of as a component of a benchmarking protocol of software for microscopy-based time-lapse analysis of single-cell growth and fluorescent protein expression. Another application of the theory is to exploit it to estimate distributions of growth characteristics that were not measured, as we outlined in the results section.

One of the limitations of the microscopic growth theory is that it is a phenomenological theory, its variables concern systemic, cellular properties and not molecular ones - i.e. like the ideal gas law in physics theory. However, we know from experiments that molecular fluctuations induce growth rate fluctuations that in turn influence molecular fluctuations^4^. A stochastic theory that incorporates this reverberating relation would be a logical next achievement. A related theoretical challenge is to extend the theory with non-steady state behaviour. For instance, to further elaborate on the Collins and Richmond’s theory^29^, which addresses growth rates of cells as function of their length, and incorporate periodic growth behaviour, such as the non-autonomous oscillatory growth rates that have been observed by Martins *et al*.^40^ with cyanobacteria. Finally, the incorporation of the mechanisms associated with size homeostasis during balanced growth^41^ would be a timely extension. These examples highlight the importance of future theoretical work that addresses some of universal aspects of balanced growth of microbial cells. However, this is not an easy challenge; the molecular complexity associated with cellular growth may prevent us from making general universal theoretical statements. Here also, the comparison with the ideal gas law fails, this law can be shown to emerge from the independent erratic motion of individual molecules; this independence makes the statistical physics theory possible, a luxury we do not have when we aim to relate molecular and cellular biology.

The microscopic growth theory therefore describes the stochasticity of the growth of symmetrically dividing single cells, it does not deal explicitly with the stochasticity of molecular processes inside cells. Although pioneering studies on molecular networks followed shortly after^42^, the coupling between those two types of theories is a much more recent development (e.g^6,22,43,44^.). Current analytical theories of the two-way coupling between single-cell growth and molecular circuit dynamics (e.g^22,42,44^.), generally deal with small circuits. Numerical simulations are therefore indispensable for making predictions in single-cell physiology. Here we provide a novel demonstration of the microscopic theory as a means to speed up stochastic simulations of the growth of a single cell and of the dynamics of complex molecular circuits inside it.

Our algorithm simulates only a single lineage, from which we can retrieve the statistics of the entire lineage tree, which is what one generally obtains with real-time imaging of single cell growth. The advantage of this algorithm is that it is fast and in agreement with the microscopic theory of single cell growth. We validated the performance of this algorithm on experimental data and found that it is performing accurately. The current limitation of this simulation algorithm is that the molecular circuit cannot influence the growth behaviour of single cells. It simulates the dynamics of a molecular circuit on which we impose the stochasticity of single cells, which grow balanced, according to the microscopic growth theory, implemented in StochPy^23^.

Even though the coupled stochastic simulation of growing cells and molecular circuits has been done (e.g^43^ and^5^), we are not aware of any software package for stochastic simulation that has this capability. StochPy is a flexible package, coded in the Python programming language. StochPy has basic stochastic simulation algorithms (i.e. of the ‘Gillespie-type’), is readily extendible by the user, uses command-line instructions, allows for coding and saving of models in scripts, has a suite of statistical analysis and plotting tools, is compliant with SBML and can exchange models with the multi-purpose, deterministic modeling software package PySCeS^45^ (http://pysces.sourceforge.net) for systems biology.

Single-cell physiology has a big impact on cell biology. Its main study focus is the heterogeneity of isogenic cell populations, due to stochasticity at the level of growing cells and their molecular circuits, its origins and physiological consequences. The stochasticity of the growth and molecular circuits of single cells, and the nonlinear effects in those circuits, makes predicting the behaviour of single cells very complicated. We therefore have to rely on theory and simulations to interpret and predict the outcome of experiments that measure the physiology of single cells. This paper contributes some of the methodology required for a quantitative and predictive single-cell physiology.

## Methods

### Microscopy experiments

#### Strain, medium and culturing

*Escherichia coli* MG1655 derived MUK21 (see^46^ for details) (kindly provided by D. Kiviet), containing a genome integrated GFP gene under the control of the wild-type lac promoter, was revived from glycerol stock by inoculating directly into M9 minimal medium (42.2 mM Na_2_HPO_4_, 22 mM KH_2_PO_4_, 8.5 mM NaCl, 11.3 mM (NH_4_)_2_SO_4_, 2.0 mM MgSO4, 0.1 mM CaCl_2_), supplemented with trace elements (63*μ*M ZnSO_4_, 70*μ*M CuCl_2_, 71*μ*M MnSO_4_, 76*μ*M CoCl_2_, 0.6*μ*M FeCl_3_), 0.2 mM uracil (all chemicals from Sigma) and 1 mM glucose as carbon source (M9-Glu). At intervals of 3 hours, the pre-culture was transferred twice to fresh M9 containing 10 mM lactose as carbon source (M9-Lac) and 1 mM of IPTG (M9-Lac-I), before inoculating an overnight culture to a final optical density (OD, 600 nm) of 2.5 × 10^−7^ in M9-Lac-I. After 16 hours, the culture was again diluted to an OD600 of 0.0025. When the culture reached an OD600 of 0.01, 2*μ*L was transferred to a 1.5% low melt agarose pad freshly prepared with M9-Lac-I.

*Bacillus subtilis* strain B1115 (amyE::Phyper-spank-sfGFP, Spcr) was constructed from parental strain BSB1 168 *trp* + using pDR111 (kindly provided by D. Rudner). B1115 was revived from glycerol stocks by directly inoculating into MM minimal medium (40 mM MOPS, 1.23 mM K_2_HPO_4_, 0.77 mM KH_2_PO_4_, 15 mM (NH_4_)_2_SO_4_, 0.8 mM MgSO_4_), supplemented with trace elements (80 nM MnCl_2_, 5*μ*M FeCl_3_, 10 nM ZnCl_2_, 30 nM CoCl_2_, 10 nM CuSO_4_), 5 mM glucose and 1 mM IPTG (MM-Glu-I) to a final OD of 4.5 × 10^−7^. After incubation for 16 hours, the culture was diluted in TSS minimal medium (37.4 mM NH_4_Cl, 1.5 mM K_2_HPO_4_, 49.5 mM TRIS, 1 mM MgSO_4_, 0.004% FeCl_3_, 0.004% Na_3_-citrate.2 H_2_O), supplemented with trace elements, 5 mM glucose and 1 mM IPTG (TSS-Glu-I) to an OD of 0.001. When the culture reached an OD600 of 0.02, 2*μ*L was transferred to a 1.5% agarose pad freshly prepared with TSS-Glu-I.

For both, *E. coli* and *B. subtilis*, pre-culturing steps served to eliminate glycerol from the medium and to ensure that the population exhibited steady-state (balanced) growth before commencing microscopy measurements.

All cultures were incubated at 37 °C, shaking at 200 rpm. Once seeded with cells, agarose pads were inverted and placed onto a glass bottom microwell dish (35 mm dish, 14 mm microwell, No. 1.5 coverglass) (Matek, USA), which was sealed with parafilm and immediately taken to the microscope for time-lapse imaging.

#### Microscopy and data analysis

Imaging was performed with a Nikon Ti-E inverted microscope (Nikon, Japan) equipped with 100X oil objective (Nikon, CFI Plan Apo *λ* NA 1.45 WD 0.13), Zyla 5.5 sCmos camera (Andor, UK), brightfield LED light source (CoolLED pE-100), fluorescence LED light source (Lumencor, SOLA light engine), GFP filter set (Nikon Epi-Fl Filter Cube GFP-B), computer controlled shutters, automated stage and incubation chamber for temperature control. Temperature was set to 37 °C at least three hours prior to starting an experiment. Nikon NIS-Elements AR software was used to control the microscope.

Brightfield images (80 ms exposure time at 3.2% power) were acquired every minute for 8 hours. GFP fluorescence images (1 second exposure at 25% power) were acquired every 10 min. Time-lapse data were processed with custom MATLAB functions developed within our group. Briefly, an automated pipeline segmented every image, identifying individual cells and calculating their spatial features. Segmentation was performed using a combination of Random Forest pixel classification and Watershed transforms (see S1 for a summary of the segmentation pipeline). Cells were assigned unique identifiers and were tracked in time, allowing for the calculation of time-dependent properties including cell ages, cell sizes (areas, lengths and widths), elongation rates and generation times. In addition, the genealogy of every cell was recorded.

Only cells from within the exponential population growth phase (see Fig. S2) were considered in the analysis. For cells expressing a fluorescent construct, the fluorescence at birth and division were taken as the first and last measurement during the single cell time trace. Because we measured the GFP fluorescence once every ten minutes, an average deviation of five minutes from *a* = 0 and *a* = *T* is present in the fluorescence data.

### Stochastic simulations

Stochastic simulations were done with the direct method algorithm^47^ extended with cell growth and division, as implemented in the CellDivision module of the stochastic simulation software package StochPy^23^. Each stochastic simulation was continued for 10^4^ generations Table 1. More information on installing and using StochPy can be found in the *StochPy User’s Guide* which together with additional example sessions is available online at http://stochpy.s f.net.

**Table 1:** Parameters used for simulating the experimental data.

Parameter	*B. subtilis*	*E. coli*
*k*_*syn*_ (min^−1^)	1.33	0.46
*n*_0_ (# molecules)	99	43
*μ* (min^−1^)	0.0099	0.0082
*L*_0_ (*μm*)	2.35	1.55
${\ell }_{D}$ (*μm*)	${\mathscr{N}}$(4.7, 0.56)	${\mathscr{N}}$(3.11, 0.30)
*ρ*	${\mathscr{N}}$(0.5, 0.033)	${\mathscr{N}}$(0.5, 0.033)

We simulated both SSA with cell growth and division for 10^4^ generations.

## Electronic supplementary material

Supplemental information

## References

[1] Balazsi G, van Oudenaarden A, Collins JJ (2011). Cellular decision making and biological noise: from microbes to mammals. Cell.

[2] Eldar A, Elowitz MB (2010). Functional roles for noise in genetic circuits. Nature.

[3] Bruggeman, F. J., Blüthgen, N. & Westerhoff, H. V. Noise management by molecular networks. *PLoS Comput Biol* **5**, 10.1371/journal.pcbi.1000506 (2009). 10.1371/journal.pcbi.1000506 PMC2731877 19763166

[4] Kiviet DJ (2014). Stochasticity of metabolism and growth at the single-cell level. Nature.

[5] Taheri-Araghi S (2015). Cell-size control and homeostasis in bacteria. Current Biology.

[6] Huh D, Paulsson J (2011). Random partitioning of molecules at cell division. Proc. Natl. Acad. Sci. USA.

[7] Kempe H, Schwabe A, Cremazy F, Verschure PJ, Bruggeman FJ (2015). The volumes and transcript counts of single cells reveal concentration homeostasis and capture biological noise. Mol. Biol. Cell.

[8] Golding I, Paulsson J, Zawilski SM, Cox EC (2005). Real-time kinetics of gene activity in individual bacteria. Cell.

[9] Boulineau S (2013). Single-cell dynamics reveals sustained growth during diauxic shifts. PLoS ONE.

[10] Schwabe, A. & Bruggeman, F. J. Single yeast cells vary in transcription activity not in delay time after a metabolic shift. *Nat Commun* **5** (2014). 10.1038/ncomms5798 25178355

[11] Kotte O, Volkmer B, Radzikowski JL, Heinemann M (2014). Phenotypic bistability in escherichia coli’s central carbon metabolis. m. Molecular Systems Biology.

[12] Solopova A (2014). Bet-hedging during bacterial diauxic shift. Proceedings of the National Academy of Sciences.

[13] van Heerden, J. H. *et al*. Lost in transition: Start-up of glycolysis yields subpopulations of nongrowing cells. *Science* **343** (2014). 10.1126/science.1245114 24436182

[14] Balaban N, Merrin J, Chait R, Kowalik L, Leibler S (2004). Bacterial persistence as a phenotypic switch. Science.

[15] Choi PJ, Cai L, Frieda K, Xie XS (2008). A stochastic single-molecule event triggers phenotype switching of a bacterial cell. Science.

[16] Veening J-W (2008). Bet-hedging and epigenetic inheritance in bacterial cell development. Proceedings of the National Academy of Sciences.

[17] Taheri-Araghi, S., Brown, S. D., Sauls, J. T., McIntosh, D. B. & Jun, S. Single-cell physiology. *Biophysics* **44** (2015). 10.1146/annurev-biophys-060414-034236 PMC5672912 25747591

[18] Delvigne F, Zune Q, Lara AR, Al-Soud W, Sørensen SJ (2014). Metabolic variability in bioprocessing: implications of microbial phenotypic heterogeneity. Trends in Biotechnology.

[19] Dhar, N., McKinney, J. & Manina, G. Phenotypic Heterogeneity in Mycobacterium tuberculosis. *Microbiology Spectrum* **4**, 10.1128/microbiolspec.TBTB2-0021-2016 (2016). 10.1128/microbiolspec.TBTB2-0021-2016 27837741

[20] El Meouche I, Siu Y, Dunlop MJ (2016). Stochastic expression of a multiple antibiotic resistance activator confers transient resistance in single cells. Scientific Reports.

[21] Paulsson J (2004). Summing up the noise in gene networks. Nature.

[22] Schwabe A, Bruggeman FJ (2014). Contributions of cell growth and biochemical reactions to nongenetic variability of cells. Biophys. J..

[23] Maarleveld TR, Olivier BG, Bruggeman FJ (2013). StochPy: a comprehensive, user-friendly tool for simulating stochastic biological processes. PLoS ONE.

[24] Schaechter M, MaalØe O, Kjeldgaard NO (1958). Dependency on medium and temperature of cell size and chemical composition during balanced growth of salmonella typhimurium. Microbiology.

[25] Monod J (1949). The growth of bacterial cultures. Annual Reviews in Microbiology.

[26] Pirt S (1965). The maintenance energy of bacteria in growing cultures. Proceedings of the Royal Society of London B: Biological Sciences.

[27] Painter PR, Marr AG (1968). Mathematics of microbial populations. Annu. Rev. Microbiol..

[28] Powell EO (1964). A note on koch and schaechters hypothesis about growth and fission of bacteria. Microbiology.

[29] Collins JF, Richmond MH (1962). Rate of growth of Bacillus cereus between divisions. J. Gen. Microbiol..

[30] Powell EO (1956). Growth rate and generation time of bacteria, with special reference to continuous culture. Microbiology.

[31] Painter PR, Marr AG (1967). Inequality of mean interdivision time and doubling time. Microbiology.

[32] Osella M, Nugent E, Lagomarsino MC (2014). Concerted control of escherichia coli cell division. Proceedings of the National Academy of Sciences.

[33] Kennard AS (2016). Individuality and universality in the growth-division laws of single e. coli cells. Physical Review E.

[34] Harvey RJ, Marr AG, Painter PR (1967). Kinetics of growth of individual cells of Escherichia coli and Azotobacter agilis. J. Bacteriol..

[35] Schaechter M, Williamson JP, Jun JH, Koch AL (1962). Growth, cell and nuclear divisions in some bacteria. Microbiology.

[36] Wallden M, Fange D, Lundius EG, Baltekin Ö, Elf J (2016). The synchronization of replication and division cycles in individual e. coli cells. Cell.

[37] Walker N, Nghe P, Tans SJ (2016). Generation and filtering of gene expression noise by the bacterial cell cycle. BMC biology.

[38] Hinkley DV (1969). On the ratio of two correlated normal random variables. Biometrika.

[39] Jun S, Taheri-Araghi S (2015). Cell-size maintenance: universal strategy revealed. Trends in Microbiology.

[40] Martins BM, Das AK, Antunes L, Locke JC (2016). Frequency doubling in the cyanobacterial circadian clock. Molecular Systems Biology.

[41] Sauls JT, Li D, Jun S (2016). Adder and a coarse-grained approach to cell size homeostasis in bacteria. Current Opinion in Cell Biology.

[42] Berg OG (1978). A model for the statistical fluctuations of protein numbers in a microbial population. Journal of Theoretical Biology.

[43] Gomez D, Marathe R, Bierbaum V, Klumpp S (2014). Modeling stochastic gene expression in growing cells. J. Theor. Biol..

[44] Marathe R, Bierbaum V, Gomez D, Klumpp S (2012). Deterministic and Stochastic Descriptions of Gene Expression Dynamics. Journal of Statistical Physics.

[45] Olivier BG, Rohwer JM, Hofmeyr J-HS (2005). Modelling cellular systems with pysces. Bioinformatics.

[46] Ozbudak EM, Thattai M, Lim HN, Shraiman BI, Van Oudenaarden A (2004). Multistability in the lactose utilization network of escherichia coli. Nature.

[47] Gillespie DT (1976). A general method for numerically simulating the stochastic time evolution of coupled chemical reactions. Journal of Computational Physics.

