# Supplementary material for: Statistics and simulation of growth of single bacterial cells: illustrations with B. subtilis and E. coli
Source: Sci Rep. 2017 Nov 23;7:16094. doi: 10.1038/s41598-017-15895-4 (PMC5700928; doi:10.1038/s41598-017-15895-4)
Supplement: Supplementary file 1 — Supplemental information [file 41598_2017_15895_MOESM1_ESM.pdf]

Supporting Information of "Statistics and simulation of growth of single bacterial cells: illustrations with *B. subtilis* and *E. coli*"

Johan H. van Heerden[1], Hermannus Kempe[2], Anne Doerr[1,3], Timo Maarleveld[1,4], Niclas Nordholt[1], Frank J. Bruggeman[1]

1. Systems Bioinformatics, VU University, De Boelelaan 1087, 1081 HV, Amsterdam, The Netherlands
2. Swammerdam Institute for Life Sciences, University of Amsterdam, Science Park 904, 1098 XH Amsterdam, The Netherlands
3. Current affiliation: Department of Bionanoscience, Kavli Institute of Nanoscience, TU Delft, Delft, The Netherlands
4. Current affiliation: Central Risk Management, ABN AMRO NV, Amsterdam, The Netherlands

Contents

1 Overview of segmentation and tracking pipeline

2 Confirmation of balanced growth during the microscopy experiment

3 Cell length and fluorescence data of a balanced growing *E. coli* population

4 The cell-size homeostasis mechanism as evident from our data

5 Cell length as an approximation for cell volume

6 Growth rate of *B. subtilis* and *E. coli* along the cell cycle, in terms of cell length and cell age

7 Fluorescence concentration and fluorescence noise for *B. subtilis* and *E. coli* along the cell cycle, in terms of cell age

8 StochPY extended with cell growth and division

8.1 Stochastic simulation of single-cell growth and gene expression matches theory . . . .

8.2 Conditions to interrelate a lineage and full tree simulation . . . .

8.3 The joint distribution of cell age and generation time . . . .

8.4 Illustrations of StochPy simulations with cell growth and division . . . .

8.5 Comparing StochPy simulations with cell growth and division to analytical solutions . . . .

Case Study 1: Volume statistics are independent of the model • Analytical solutions: Case Study 1 Volume distributions • Case Study 2: mRNA synthesis • Analytical solutions: Case Study 2 mRNA synthesis • Poisson distributed molecule copy numbers at a specific age • mRNA copy number distribution of a sample of extant cells • Case Study 3: mRNA synthesis and degradation • Analytical solutions: Case Study 3 mRNA synthesis and degradation • Poisson distributed molecule copy numbers at a specific age • Molecule copy-number distribution of a sample of extant cells

1 Overview of segmentation and tracking pipeline

In order to extract growth and protein expression dynamics from time-lapse microscopy experiments, we developed a custom segmentation and cell tracking pipeline. Figure S1 shows an overview of this pipeline. Briefly, for each frame of the experiment, we use the Fiji[48] implementation of the Trainable Weka Segmentation plugin[49] to generate initial segmentations of cell objects using the Random Forest classification algorithm (Fig. S1A). This initial segmentation is read into Matlab (R2015b) as a mask for further refinements using a Watershed transform. Next, unique identifiers are assigned to each segmented object and tracked in time. Tracking is performed for each frame immediately after segmentation, as this allows information from the previous frame to be used in cell identifier assignment (Fig. S1B). From the time-dependent contours of individually segmented cells, all features including size, age, genealogy and fluorescence can be obtained (Fig. S1C).

2 Confirmation of balanced growth during the microscopy experiment

During steady state balanced growth, the specific growth rate and other properties of the population should remain fixed for a duration longer than several generation times[27]. We confirmed this by plotting the logarithm of the total cell length of the population as function of time and identifying a linear region, during which balanced growth occurs (Fig. S2).

During balanced growth, the total fluorescence of cells, which corresponds to the levels of a constitutively expressed protein, should increase exponentially with time (Fig. S2). This indicates that the protein abundance changes in proportion to the total cell volume, which in our case simplifies to proportional increase with cell length, as the cell maintain a fixed cell width (Fig. S3). That the concentration of a constitutively expressed protein remains fixed during balanced growth can be deduced from the differential equation of the concentration, denoted by c, which equals the ratio of the protein copy number n (assumed proportional to protein fluorescence) and the cell volume, V,

$$\frac{dc}{dt} = \frac{d}{dt}\frac{n}{V} = \frac{1}{V}\frac{dn}{dt} - \frac{n}{V^2}\frac{dV}{dt}$$

When concentration is at steady state then:

$$\frac{1}{V}\frac{dn}{dt} = \frac{n}{V^2}\frac{dV}{dt}$$

We can now identify the specific growth rate from,

$$\frac{1}{n}\frac{dn}{dt} = \frac{1}{V}\frac{dV}{dt} := \text{specific growth rate of the population,}$$

indicating that n and V increase exponentially in time. From this we can conclude that during exponential growth the concentration of the protein remains fixed, this implies that the protein copy number increases exponentially at the same rate as the cell volume.

Another requirement of balanced growth is that the probability distributions of growth characteristics are invariant with time, which is illustrated by Fig. S4.

3 Cell length and fluorescence data of a balanced growing *E. coli* population

In addition to the *B. subtilis* data, which we presented in the main text, we measured cell growth characteristics (Fig. S5) and gene expression (Fig. S6) in a balanced-growing *E. coli* population. As for *B. subtilis*, the *E. coli* experimental data shows a remarkable agreement with the microscopic growth theory and stochastic simulations.

4 The cell-size homeostasis mechanism as evident from our data

A single-cell growth characteristic that has since recently received a lot of attention is the mechanisms which cells use to achieve size homeostasis during balanced growth[5]. Since the probability distributions for cell size are constant during balanced growth, cells that are either smaller or larger than the average cells size compensate for their size discrepancies. At least three mechanisms have been proposed that lead to cell-size homeostasis[5]. Cells can either be 'sizers', 'adders' or 'timers', or they follow one of two mixed mechanisms: 'sizer-like adder' or 'timer-like adder'[39].

Sizer, adder and timer mechanisms can be distinguished from the slope of the relation between the average length that cell adds during a single generation and its birth length (i.e. $\langle \Delta l | l_B \rangle$ as function of l_B)[5,39,41]. The slopes we find in the $\langle \Delta l | l_B \rangle$-vs-$l_B$ plot (Fig. S7) are slightly negative (≈ -0.3), indicating that both *B. subtilis* and *E. coli* follow a sizer-like adder mechanism.

5 Cell length as an approximation for cell volume

During balanced growth, cell volume (and cell length when the width remains fixed) double each cell cycle. On average, the mean volume at division is twice the mean birth volume. We measure cell growth with a time resolution of one minute, the determined moment of cell birth and cell division deviates on average by half a minute. Within this time window minor cell growth and deformation changes might occur, disturbing the expected $\langle l_B \rangle = \frac{1}{2} \langle l_D \rangle$ relation. Indeed, the observed cell length which we use as a measure for cell volume, does not double exactly in length during a cell cycle. When we use the combined length of both daughter cells as measure for division length the expected $\langle l_B \rangle = \frac{1}{2} \langle l_D \rangle$ relation holds.

Since the deviation in cell length is more than expected in a minute of cells growth, we consider the influence of rod-shaped bacterial growth to bring forward a possible explanation. When the exact moment of cell division is not determined perfectly, the mother cell might represent more of a single rod shape rather than two rods (Fig. S9). This figure indicates that a single cell can double its volume while not doubling its length.

To estimate the maximal deviation between volume and length growth, we consider cells as perfect rod-shaped until the moment of division. We observed a constant cell width during a cell cycle (Fig. S3), hence the radius of the cell is fixed. The difference in cell length and volume growth is caused by the cell poles, which are rounded. The in length increase to double the volume of a rod is $2L_b - \alpha$, in which α is given by:

$$\begin{aligned} \pi r^2(2r - \alpha) &= \frac{4}{3}\pi r^3 \\ \alpha &= \frac{2}{3} r \end{aligned} \tag{S1}$$

this solves the cylindrical height to equalise the volume of a sphere (the sum of the two rounded poles) to a cylinder. This means that the deviation in observed cell length growth can be at max $\frac{2}{3}r$. In table S1 we provide this minimal estimate of cell length compared to the measured cell length for L_B, L_D, and ΔL. The measured mean lengths fall somewhere between exact doubling of cell length and the calculated length increase based on a rod-shaped cell throughout the entire cell cycle. Given the length statistics of *B. subtilis* we predicts a minimal ΔL of 2.19 μm. For the *E. coli* data the predicted minimal ΔL is 1.43 μm. It is known that *B. subtilis* and *E. coli* divide using different division modes[50]. In *E. coli* the cell membrane grows inwards while *B. subtilis* assembles a septal wall. Due to this differences in division mechanisms, the ΔL of *E. coli* might be more close to L_B. While the ΔL of *B. subtilis* is more close to discussed rod-shaped mother cells. Of interest, using the combined length of both daughter cells as measure for division length, gives $\langle \Delta L \rangle = 2.50$ for *B. subtilis* and $\langle \Delta L \rangle = 1.62$ for *E. coli*.

	B. subtilis			*E. coli*		
	L_B	ΔL	L_D	L_B	ΔL	L_D
min		2.19	4.60		1.43	2.95
**data**	**2.46**	**2.24**	**4.70**	**1.60**	**1.51**	**3.11**
max		2.46	4.92		1.60	3.20

Table S1. Average cell length calculated from the birth length distribution (ℓ_B). The range is given by the perfect rod-shape model and the double of birth length. The data indicates that the measured length falls within the expected range.

6 Growth rate of *B. subtilis* and *E. coli* along the cell cycle, in terms of cell length and cell age

Figure S10 shows the instantaneous growth rate of *E. coli* and *B. subtilis* at particular progression extends along their cell cycle, at a particular length (Fig. S10A and C) and a particular age (Fig. S10B and D). The conditional mean growth rate, i.e. $\langle \mu | l \rangle$ and $\langle \mu | age \rangle$ is fixed along the cell cycle.

7 Fluorescence concentration and fluorescence noise for *B. subtilis* and *E. coli* along the cell cycle, in terms of cell age

The fluorescence concentration and noise hardly change as function of the progression along the cell cycle as is indicated by the slopes of the conditional mean lines as function of cell age in Fig. S11. Only the fluorescence noise data of *E. coli* shows a decrease of about 20% from birth to division.

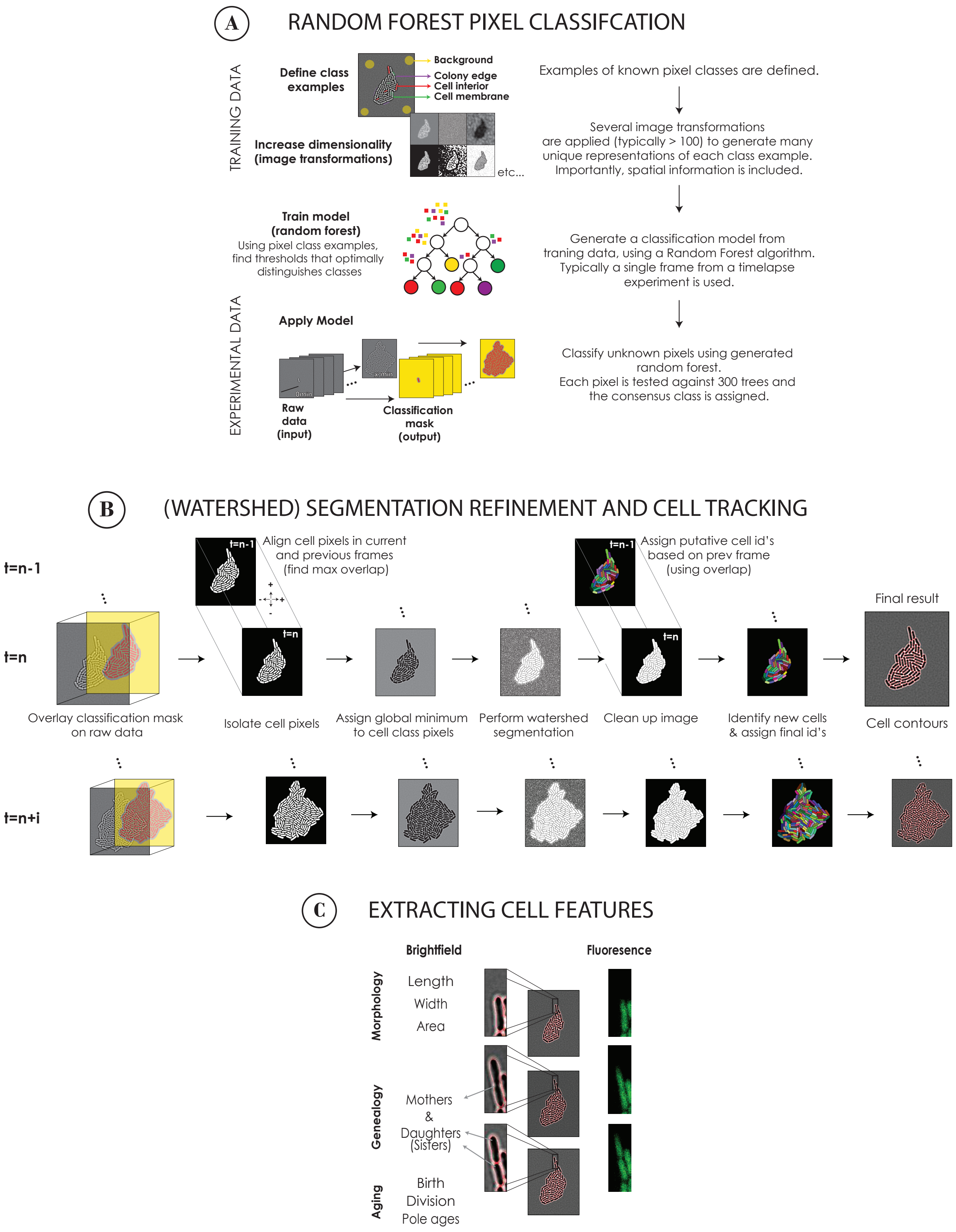

Figure S1. Overview of the segmentation and tracking pipeline. (A) Using Random Forest classification, pixels from raw images are assigned to one of four classes: background, colony edge, cell interior and cell membrane. (B) Further refinement of the initial segmentaiton is achieved by a watershed transform, using the Random Forest classified image as an input mask. Once segmentation is complete, identifiers are assigned to cell objects, using information from the current and previous frames. (C) From the contours of segmented cell objects, all spatial and temporal features can be calculated.

.

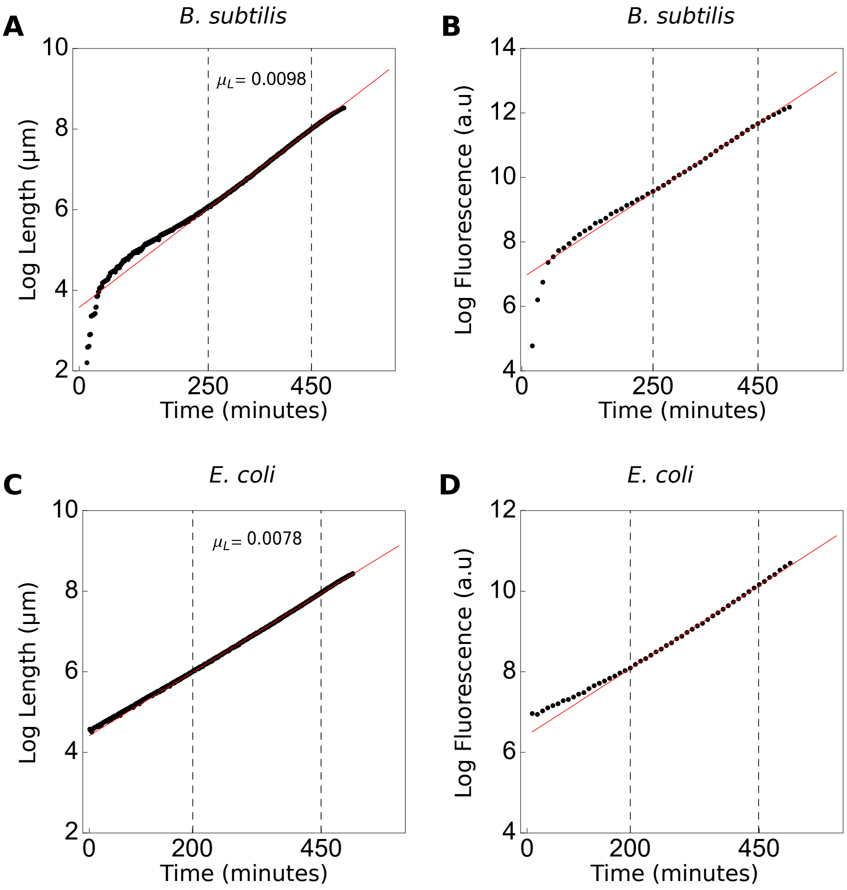

Figure S2. Linear fit of the log transformed total cell length and fluorescence of the *B. subtilis* and *E. coli* population. The total length of all individuals in the (A) *B. subtilis* population increases with 0.0098 min^{-1} and for *E. coli* this rate is 0.0078 min^{-1} (C). The dashed lines marks the region of balanced growth, i.e. where growth rate is fixed over multiple generations, used for data analyses. Similarly, the rate of total fluorescence increase is fixed during balanced growth, for both (B) *B. subtilis* (D) and *E. coli*.

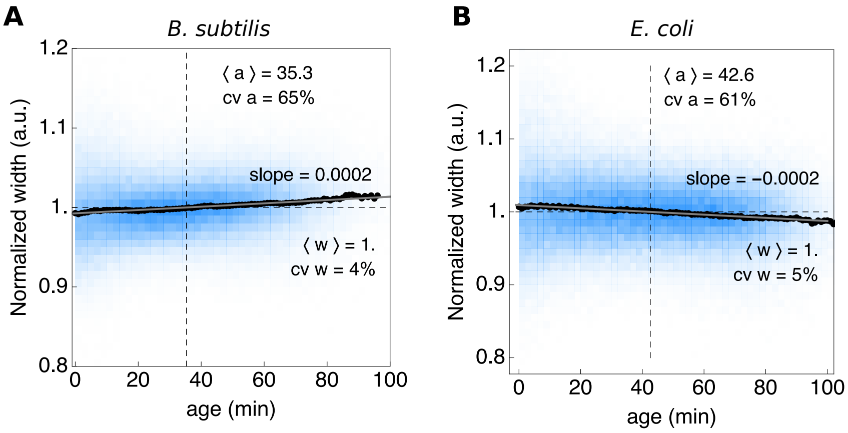

Figure S3. Cell width as function of age for a *B. subtilis* and *E. coli* population. The width of cells remain approximately constant during their life cycle.

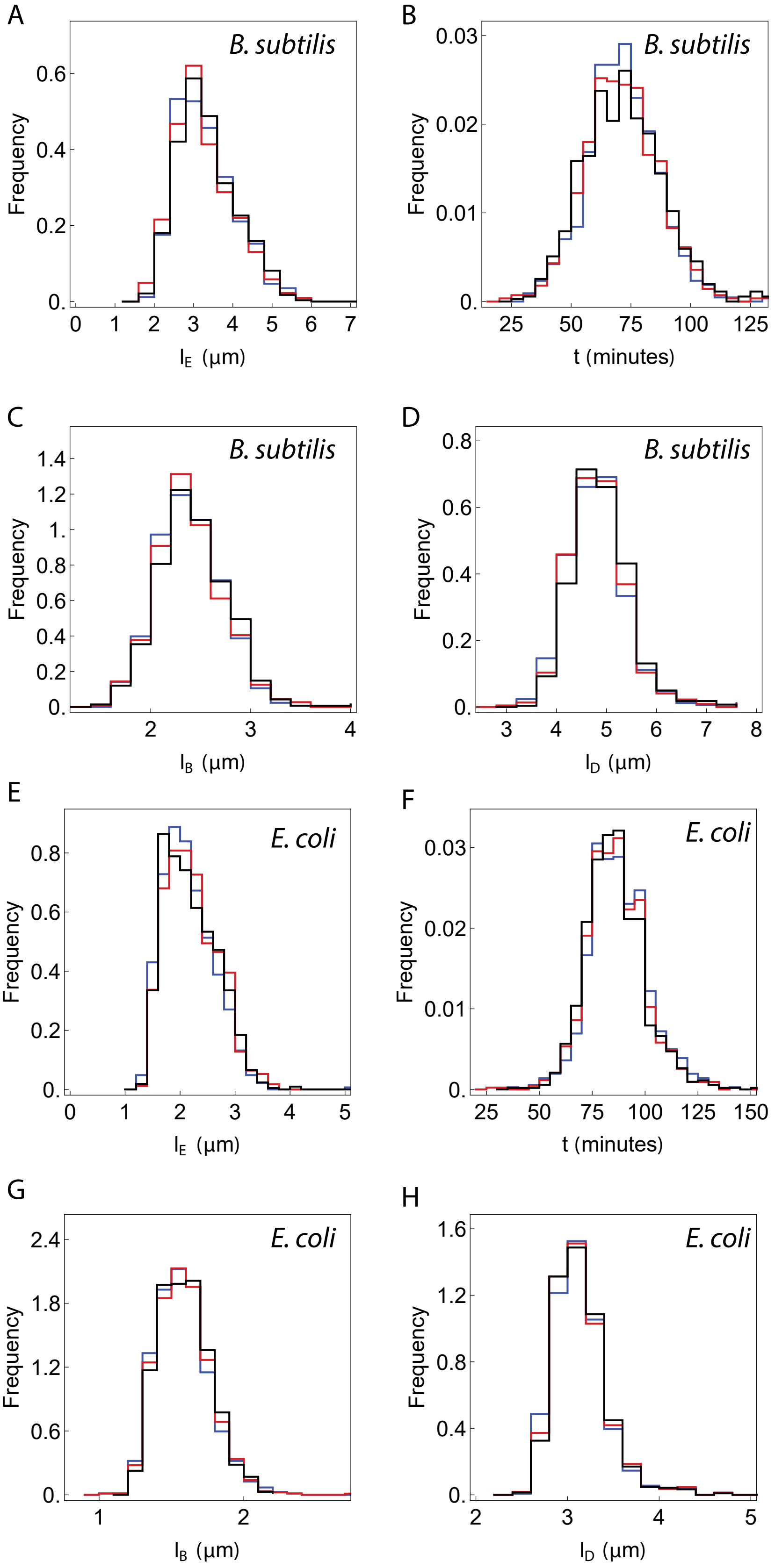

Figure S4. Balanced growth is characterised by the time-invariant distribution of growth characteristics. A defining characteristic of balanced growth is that the probabilities to observe cells with particular growth properties – their phenotype – are fixed and the associated probability distributions are therefore time invariant. The extant cell length (A and E), generation time (B and F), birth (C and G) and division length (D and H) distributions sampled at three different time points (375 min, blue; 400 min, red; 425 min, black) during balanced exponential growth (see Fig. S2) are shown for a *B. subtilis* and *E. coli* population.

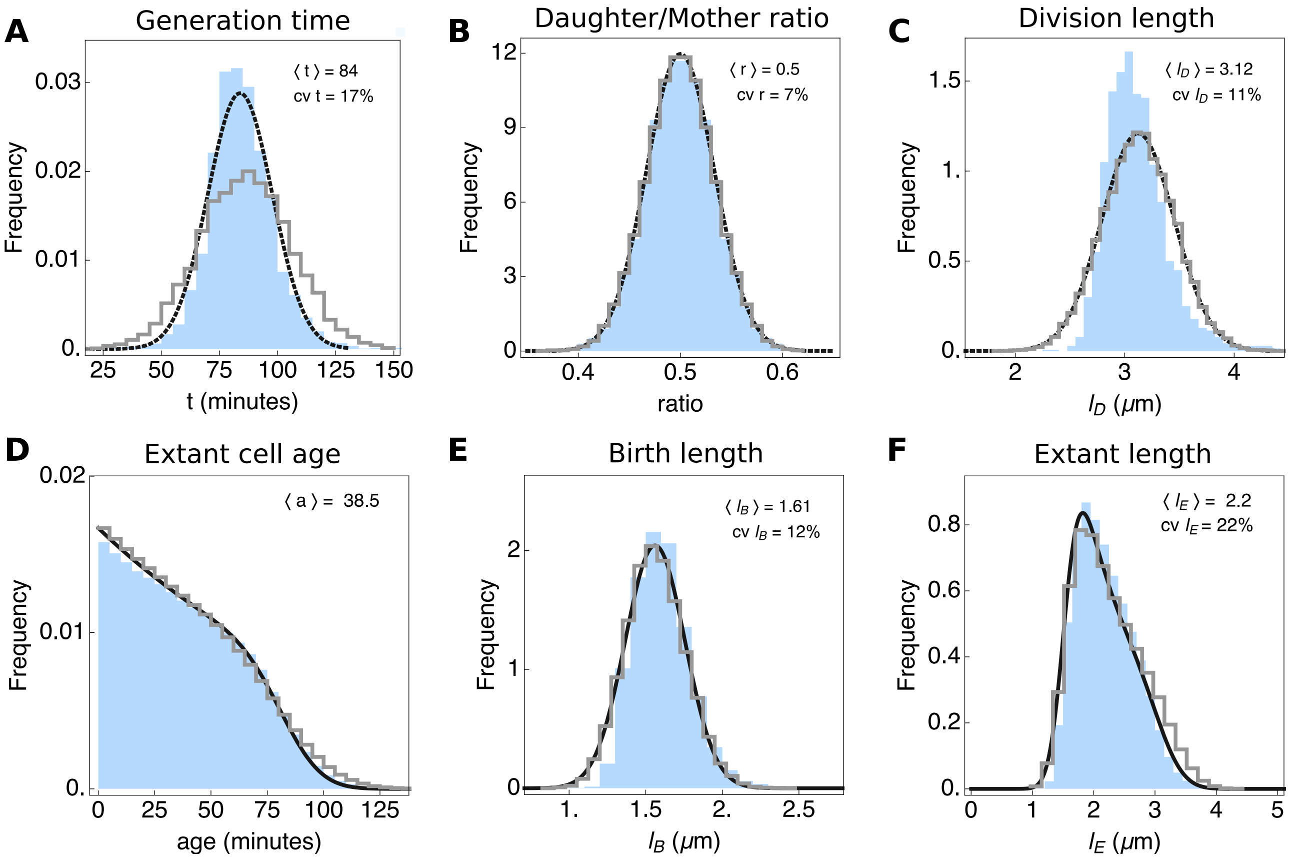

Figure S5. Growth characteristics of *E. coli*. Shown are results comparing the microscopic growth theory relations derived by Collins & Richmond[29], Powell[28] and Painter & Marr[27] and experimental single-cell growth data. This figure is the same as Fig. 2, but shows data for *E. coli*. The probability distributions obtained from experimental data are shown in blue, the predicted distributions (obtained using the relations defined in eqs. 1 - 4 in Fig. 2), are shown in black in (D), (E) and (F), and the results of our simulation algorithm are shown as grey histograms. In (A), (B) and (C), black curves indicate fits to the measured data. The sample sizes for the experimental data are 4558 extant cells, 3617 cells at birth and 1867 cells at division.

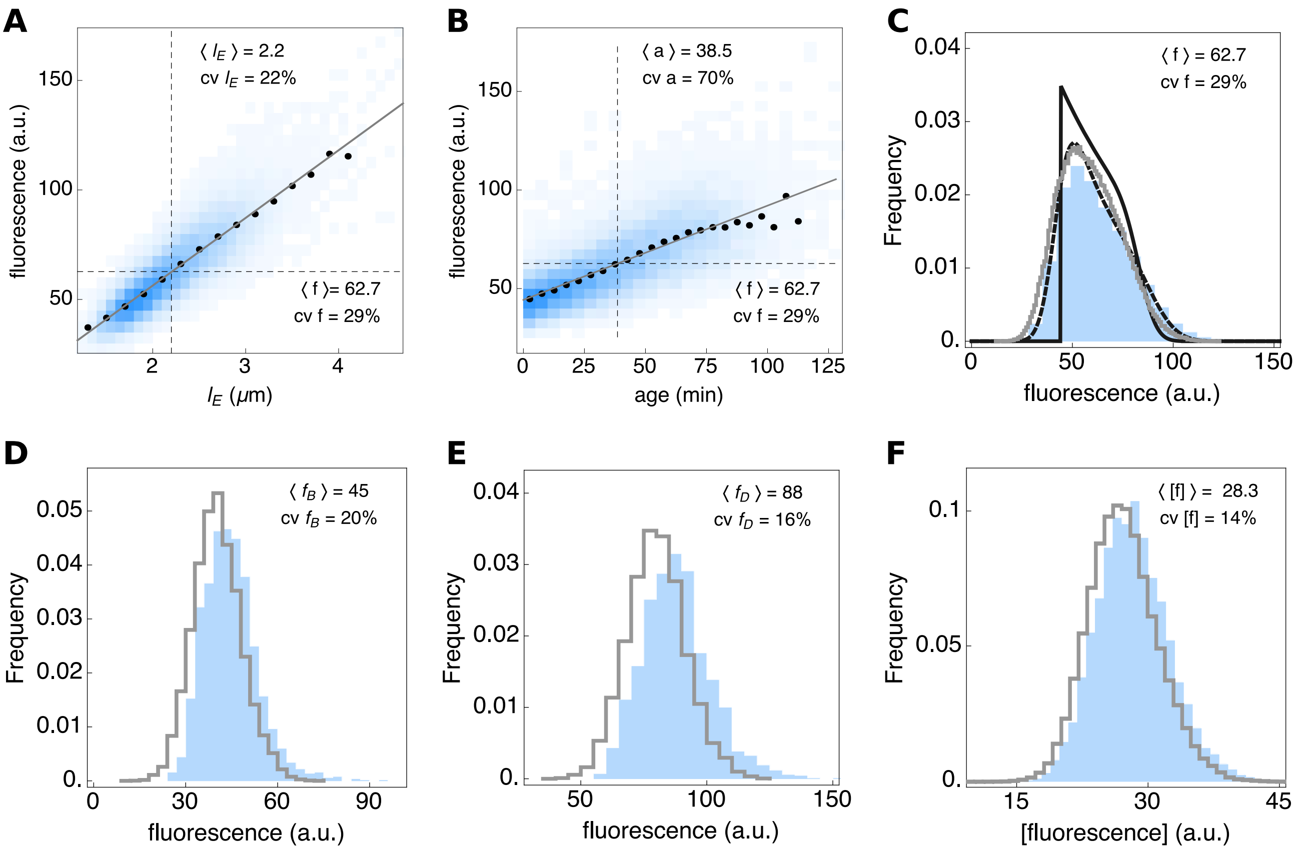

Figure S6. Expression data of the reporter gene for *E. coli*. (A) Whole-cell fluorescence as function of cell length with black dots as conditional mean $\langle f|l_E\rangle$. (B) Cell fluorescence as function of cell age with black dots as conditional mean $\langle f|age\rangle$. (C) Whole-cell fluorescence probability density: experimental data (blue), predicted fluorescence probability density obtained from the extant length distribution and the relation between fluorescence and length (shown in A) (dashed, black line), predicted fluorescence probability density obtained from the age distribution and the relation between fluorescence and age (shown in B) (black line) and the stochastic simulation (grey line). The experimentally determined distributions (blue) of whole-cell fluorescence at (D) cell birth, (E) cell division and (F) the distribution of fluorescence concentration of extant cells, is compared to simulations (grey lines).

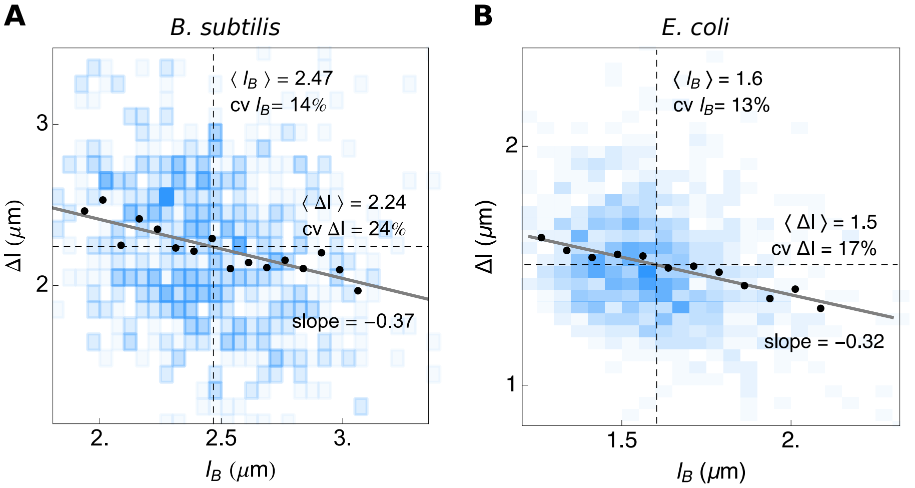

Figure S7. Cell-size homeostasis mechanisms inferred from the single-cell growth data of *B. subtilis* and *E. coli*. From the slope of the dependency of average cell length added during a single generation on the length at cell birth ($\langle \Delta l | l_b \rangle$-vs-$l_b$), the cell-size homeostasis mechanism can be inferred. The slopes of about -0.3 indicate that, under the conditions tested, (A) *B. subtilis* and (B) *E. coli* behave mostly as adders with a small sizer effect.

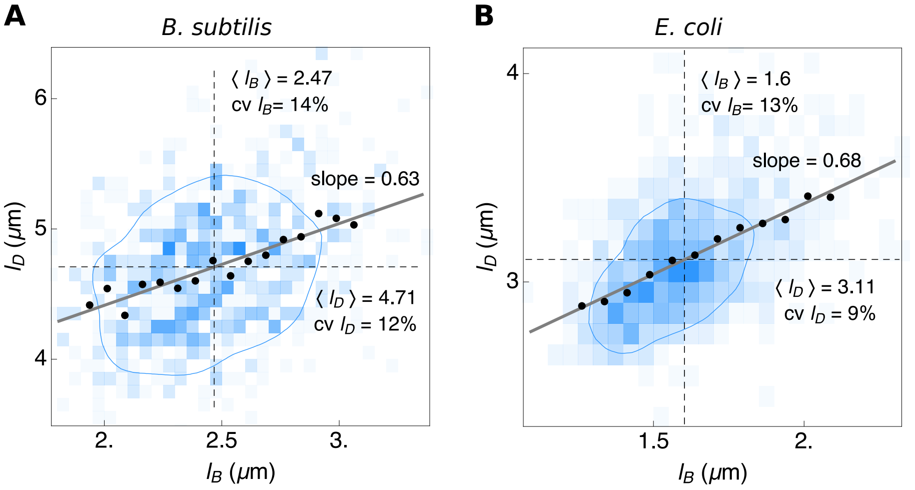

Figure S8. Relation between the cell volume at birth and division inferred from the single-cell growth data of *B. subtilis* and *E. coli*. The length at cell division as function of the length at birth ($\langle l_D | l_B \rangle$-vs-$l_B$) of (A) *B. subtilis* and (B) *E. coli* shows a weak positive correlation.

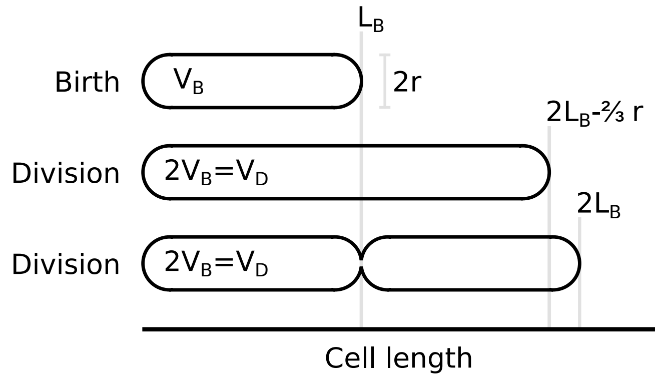

Figure S9. Cell shapes at birth and division. At cell division the average cell volume is doubled compared to the birth volume. Depending on the cell shape of the mother cell at division, the cells do not display full length doubling during a single cell cycle. The maximal deviation between volume and length increase can be calculated and is $\frac{2}{3}r$.

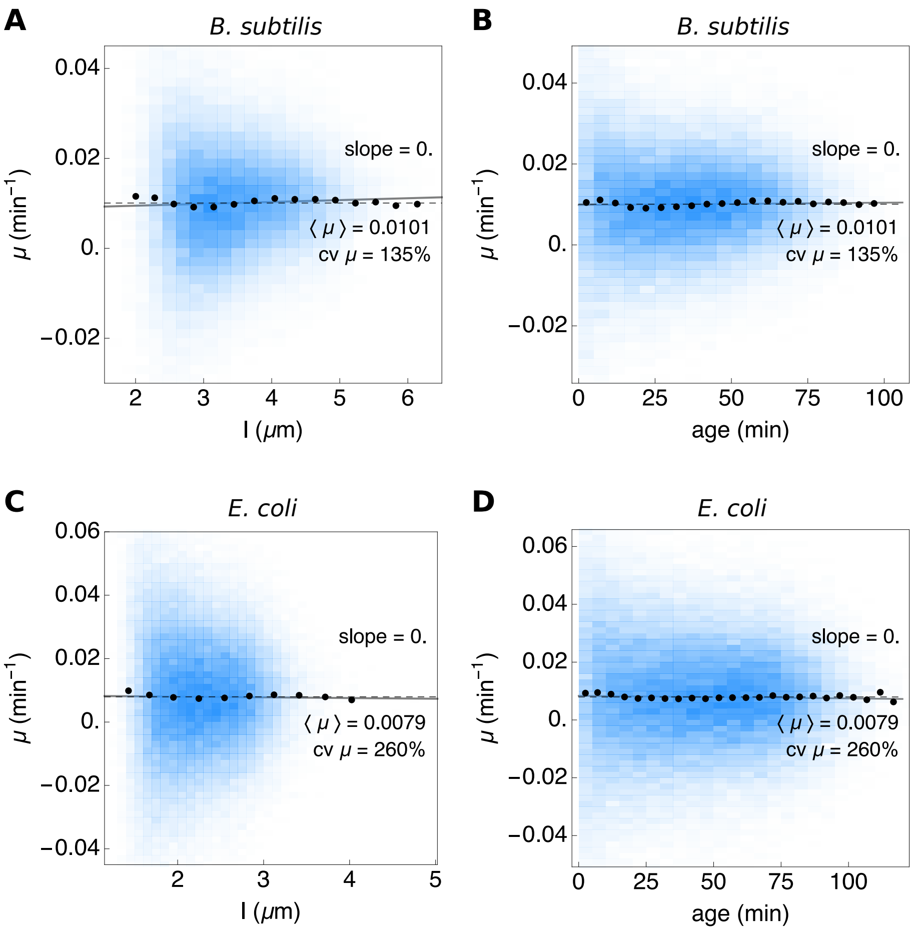

Figure S10. Instantaneous growth rate as function of age and cell length for *B. subtilis* and *E. coli*. The blue density histograms give the distribution of the instantaneous growth rate as function of length (A and C) and age (B and D). The population means are shown by the dashed black lines. A linear fit to the full dataset is shown in gray and the conditionals $\mu|l$ and $\mu|age$ by the black dots.

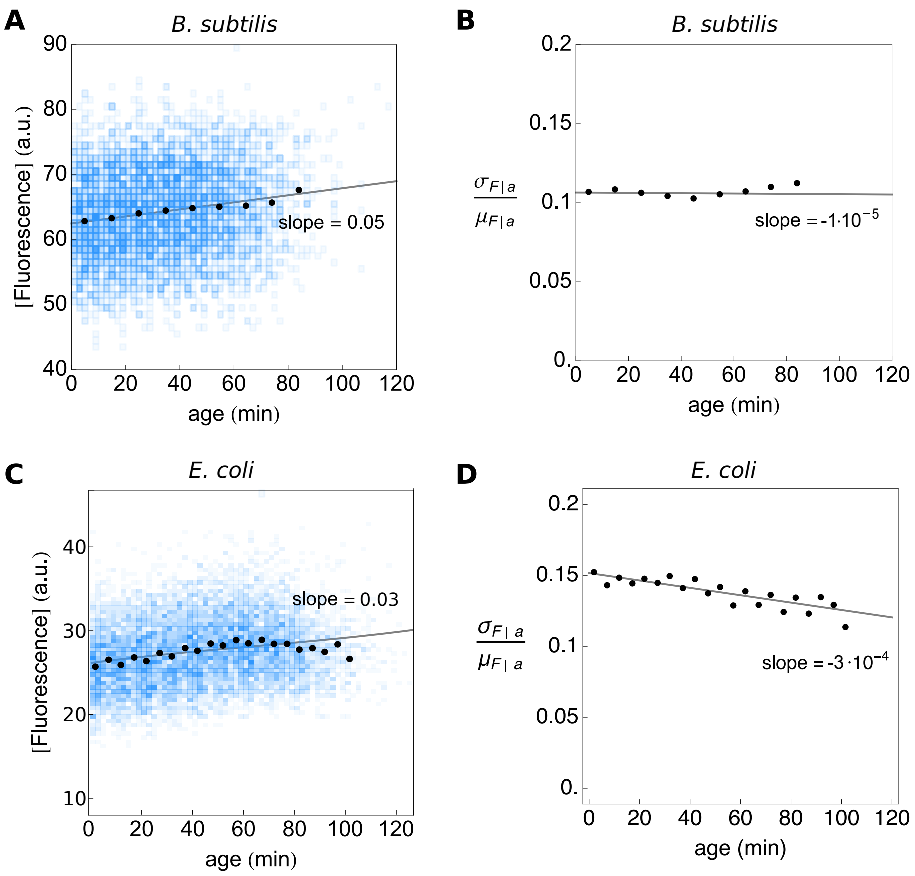

Figure S11. Concentration of fluorescence and fluorescence noise as function of age for the *B. subtilis* and *E. coli* data. The average concentration of fluorescence remains stable during a cell cycle. The cv of the concentration of fluorescence does not increase as function of age, explaining the similarity between the theoretically predicted and observed expression distribution (see Fig. 4C).

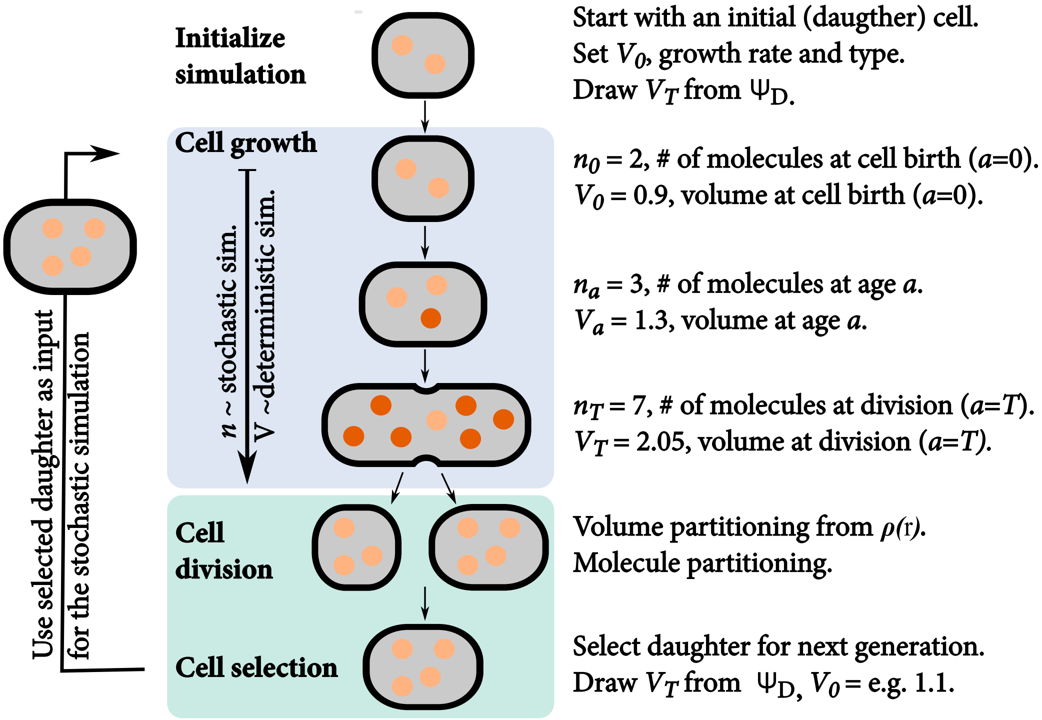

Figure S12. The SSA with cell growth and division as incorporated in StochPy. The simulation starts with one daughter cell of which we track a single lineage through time. We perform a stochastic simulation until the generation time T is reached. Then, the mother volume is partitioned between both daughter cells. Molecules are subsequently partitioned (volume dependent) between both daughters. The light molecules are inherited from the mother cell, darker ones are newly synthesised during the cell cycle. This procedure is repeated until either the number of generations is reached, the desired end time is reached, the desired number of time steps is reached, or all reactions are exhausted.

8 StochPY extended with cell growth and division

The stochastic simulation algorithm that couples the simulation of molecular circuits to that of cell growth and division is implemented in StochPy[23] and available for download from `http://stochpy.sf.net`. Figure S12 gives an overview of how we extended the SSA with cell growth and cell division.

We discuss the steps outlined in Fig. S12 step-by-step (the notations used are explained in Table S2):

1. **Initialize simulation.** In the extended SSA, containing cell growth and division, we simulate a single lineage, so we start the simulation with a single cell. Before we can perform a simulation we have to initialize the simulation by setting various parameters. For instance, we have to set the cell volume of the initial cell (V_0). A cell can be in any cell-cycle stage ($0 \leq a \leq T$) where a is the cell age and T the generation time—the time period between two consecutive cell divisions. We parameterise the initial cell to start at the beginning of the cell-cycle stage, i.e. $a = 0$. During the simulation, the cell grows deterministically, at the specified specific volume-growth rate (μ) and according to a particular type of growth—we support both exponential and linear volume growth rates. Next, we have to determine when the tracked cell divides. We do this by drawing a volume (V_T) at which the mature mother cell divides into two daughter cells. The volume at which the mother cell divides is drawn from a probability density function (PDF) Ψ_D. This PDF is independent from the volume at birth, which makes the volume at division independent from the volume at birth. Given values for V_0, V_T, μ, and the growth, the generation time T can be calculated. We illustrate this here for an exponential growth rate:

$$V_T = V_0 \cdot e^{\mu T} \rightarrow T = \frac{\ln(V_T/V_0)}{\mu}. \tag{S2}$$

2. **SSA coupled to cell growth.** Once we know T, we can start the SSA until the division event at $a = T$. Modeling cell growth implies that V increases during the simulation from birth ($a = 0$) to division ($a = T$). In a growing cell, reacting molecules require more time to find each other, thus the reaction waiting times for these kind of reactions increases. This

Table S2. Notations of used variables. For balanced, exponentially-growing cells the volume specific growth rate (μ) is equal to the specific growth rate of the population (k).

Variable	values	pdf	description
T	t	$\tau(t)$	Interdivision time
R	r	$\rho(r)$	Partitioning ratio
V			Cell volume
V_B	v_B	$\Psi_B(v_B)$	Birth volume
V_D	v_D	$\Psi_D(v_D)$	Division volume
V_E	v_E	$\Psi_E(v_E)$	Extant volume
L			Cell length
L_B	l_B	$\ell_B(l_B)$	Birth length
L_D	l_D	$\ell_D(l_D)$	Division length
L_E	l_E	$\ell_E(l_E)$	Extant length
A	a	$u(a)$	Cell age
k			Specific growth rate of the population
μ			Cell volume growth rate

means that the propensity functions of diffusion limited reactions (i.e. second and higher-order reactions) depend on V. We therefore inserted V as a variable in the respective propensity function,

$$a_{j,V(t)} = \frac{a_j(t)}{V(t)^{order-1}} \text{ with order } \geq 2. \tag{S3}$$

The propensity functions of zero and first-order reactions are unaffected by V. While we model V deterministically we only update V at each stochastic event in the simulation. This means that we calculate the time until the next reaction fires based on the V at the time of firing of the previous reaction; V is larger at the moment of reaction execution which results in a underestimation of the reaction time of second and higher-order reactions. This effect is negligible if the volume difference between the two consecutive firings, ΔV, is small. An alternative method could be to add volume as an additional reaction stochastic reaction that fires frequently as was done by[43], but this slows down the simulation.

3. **Cell division.** The SSA continues until the generation time T is reached (and $V = V_T$). Both V_T and x_T are then partitioned between the two daughter cells. The partitioning ratio is drawn from the PDF $\rho(r)$. Daughter one and two receive a volume of $V_{d1,0} = r \cdot V_T$ and $V_{d2,0} = (1-r) \cdot V_T$ respectively. The $\rho(r)$ distribution should be symmetric around a mean of 0.5, otherwise a bias for one daughter is created. The partitioning of molecules between both daughter cells—which is done next—is also a stochastic process and depends on the cell volumes of both daughters. This partitioning of molecules is, therefore, modeled with a volume-dependent binomial distribution. More specifically, the probability that a specific molecule is inherited by daughter one is modeled as $V_{d1,0}/V_T$. This means that the number of molecules, with copy number n, inherited by daughter one can be drawn as a random sample from a binomial distribution with n number of trials and success probability $V_{d1,0}/V_T$. The process is repeated for each species. Not all cellular constituents should be binomially distributed between both daughter cells. DNA is an example of this; each daughter cells receives one copy of the chromosome in a normal cell division event. These kind of cellular constituents divide exact. Stochastic simulations also allow the definition of fixed species—species that do not change in copy number of concentration over time—which are not divided during a cell division event.

4. **Cell selection.** Starting the SSA with a single cell and simulating the entire population tree is computationally difficult or impossible. We, therefore, track a single cell lineage through time, which allows us to incorporate cell growth and division in stochastic simulation algorithms in an efficient manner. While simulating a single lineage was also done by[43], we are also able to get the statistical properties of either the whole tree or a sample of extant cells from a single lineage over time. This process is explained in the following section.

8.1 Stochastic simulation of single-cell growth and gene expression matches theory

Within a population of balanced growing cells, three types of samples can be distinguished: samples of extant, mother, and baby cells; their definitions can be found in[27] and in the main text. The statistics of cell age, generation time and cell volume of these samples are interrelated, for the generation time the following relation holds:

$$\tau_e(t) = 2\left(1 - e^{-kt}\right)\tau_b(t) = \left(e^{kt} - 1\right)\tau_m(t) \tag{S4}$$

where k denotes the specific growth rate of the population and $\tau_s(t)$ gives the probability density function of the generation times for the different types of samples (with s as either e: extant, b: baby, or m: mother). During lineage simulations we select at each division the daughter cell which will be followed in such a way that the statistics of the resulting lineage corresponds to a sample of mother cells.

To generate a lineage that is representative for a sample of mother cells, at each division the daughter to be followed by the simulation is chosen with a probability according to the fraction of descendants it can be expected to contribute to the population: If daughter 1 is expected to have n_1 descendants at a later time point t_x and daughter two n_2 descendants, the probability p to choose daughter 1 is given by

$$p = \frac{n_1}{n_1 + n_2}. \tag{S5}$$

Let T_1 and T_2 be the generation times of daughters 1 and 2, respectively. In balanced growth the number of expected descendants at time t_x is then given by $n_1 = e^{k\cdot(t_x - T_1)}$ and $n_2 = e^{k\cdot(t_x - T_2)}$. This is possible because the growth law does not depend on molecule concentrations or previous history. Inserting these relationships for n_1 and n_2 in Eq. (S5) gives

$$p = \frac{e^{k\cdot(t_x - t_1)}}{e^{k\cdot(t_x - T_1)} + e^{k\cdot(t_x - T_2)}} = \frac{e^{k\cdot(T_2 - T_1)}}{1 + e^{k\cdot(T_2 - T_1)}} = \frac{e^{k\cdot\Delta T}}{1 + e^{k\cdot\Delta T}} \tag{S6}$$

where $\Delta T = T_2 - T_1$. In short, the larger daughter cell is more probable to be chosen, because this cell will reach the next division volume sooner and is therefore likely to have more descendants in the population at a later time point.

With as simulation result a lineage that represents a sample of mother cells, statistical properties of other defined samples can be calculated based on the known relationships of generation time and cell age between these samples[27].

8.2 Conditions to interrelate a lineage and full tree simulation

Here we state the conditions for the simulation of a single lineage that can be used to retrieve the statistics of the entire lineage tree:

1. **The volume at which cells divide has to be independent of the volume at birth, and any other cell-specific properties like concentrations of certain molecules etc.** To ensure that volumes at division are independent of volumes at birth, the distribution of division ratios (ρ) and the distribution of volumes at division (Ψ_D) are chosen such that the largest possible volume at birth is smaller than the smallest possible volume at division (i.e. there is no overlap between the two volume distributions). By default we use beta distributions for both the division ratio as well as the volume at division. Beta distributions are bounded and symmetric if its two positive shape parameters are identical.

2. **The growth law for a single cell has to be deterministic and independent of intracellular concentrations.** The current implementation allows exponential and linear growth laws for single cells. For an exponential growth law the volume specific growth rate (μ) is equal to the specific growth rate of the population (k). Otherwise, the specific growth rate of the population needs to be calculated. We do this by using the known relations between the volume at division, volume at birth, the growth law, the volume distribution of extant cells (Equation (S7)[27]), and the fact that this volume distribution has to integrate to one:

$$\int_0^{V_{max}} \Psi_E(V)dV = \int_0^{V_{max}} e^{-R(V)} \cdot \left(\int_0^V \frac{k \cdot e^{R(\bar{V})}[2\Psi_B(\bar{V}) - \Psi_D(\bar{V})]}{g(\bar{V})} d\bar{V} + C \right) dV = 1 \tag{S7}$$

where

$$R(\bar{V}) = \int_0^{V_{max}} \frac{(g'(\bar{V}) + k)}{g(\bar{V})} d\bar{V}. \tag{S8}$$

Here V is the cell volume, C an integration constant (which can be calculated from boundary conditions of the volume distribution), $\lambda_e(V)$ the volume distribution of a sample of extant cells, $\Psi_B(V)$ the volume distribution at birth for a sample of baby cells, $\Psi_D(V)$ the volume distribution of a sample of mother cells, and $g'(V)$ the differential of the formula of cell volume growth ($g(V) = \mu \cdot V$ for exponential growth and $g(V) = \mu$ for linear volume growth, with μ as the volume specific growth rate). We know $\Psi_D(V)$, $g(V)$, $g'(V)$, and C from the model parameters. $\Psi_B(V)$ can be calculated from $\Psi_D(V)$ and the partition distribution $\rho(r)$ using[28]:

$$\Psi_B(V) = \int_V^\infty \frac{\Psi_D(\theta)}{\theta} \cdot \rho(\frac{V}{\theta}) d\theta. \tag{S9}$$

A solution for Eq. (S7) is approximated by using the secant or Newton-Raphson method.

For example, we can calculate the copy number distributions for a sample of extant cells (corresponding to any experiment that records molecule copy numbers at a fixed moment in time as for example smFISH) from the simulation of a single lineage by using

$$p(N_x = n) = \int_0^{T_{max}} \int_0^{a_{max}} g(a,T) \frac{I(n,a,T)}{\sum_j I(j,a,T)} dadT. \tag{S10}$$

Here, $\frac{I(n,a,T)}{\sum_j I(j,a,T)}$ is the relative frequency of copy number equal to N_x in the simulated lineage at age a for a cell with generation time T approximating $p(N_x = n|a,T)$ and $g(a,T)$ is the joint PDF of cell age and generation time,

$$g(a,T) = k\tau_m(T) \cdot e^{k(T-a)}. \tag{S11}$$

The derivation of $g(a,T)$ is given in Eq. S13 and S14. Using Eq. (S11) we can rewrite Eq. S10 to:

$$p(N_x = n) = \int_0^{T_{max}} \int_0^{a_{max}} \tau_m(T) \cdot k \cdot e^{k \cdot (T-a)} \frac{I(n,a,T)}{\sum_j I(j,a,T)} \cdot dadT, \tag{S12}$$

where $I(n,a,T)$ is an indicator function equal to the number of occurrences of copy number $N_x = n$ at cell age a in a cell with generation time T. Obtaining the statistics for an extant cell population consists of two main steps. First, in order to work with the indicator functions, the simulation time series needs to be binned. The binning needs to be performed at regular intervals for cell age. Secondly, a double integral has to be taken which is a slow procedure for infinitesimal small steps of a and T. We approximate this double integral by using the Riemann sum (or the 2D trapezoidal rule).

Additionally, the statistics for a sample of extant cell volumes can be calculated from the simulation of a single lineage by re-using Eq. S12 and replacing copy numbers with volume data. Calculating the statistics for sample of extant cell volumes requires one additional step—the continuous volume data must be binned to make the distribution discrete.

8.3 The joint distribution of cell age and generation time

In order to obtain the copy number distributions for a sample of extant cells the joint distribution of cell age and generation time for extant cells is required which can be calculated knowing the growth rate and the generation time distribution. The derivation presented here is analogous to the one for the distribution of extant cell generation times in[27] (p. 528). With a population size of n_0 at $t = 0$ the rate of formation of new cells equals $2k \cdot n_0 \cdot e^{kt}$. The fraction of these cells with generation time smaller than τ which survive at least until time t is given by $\int_{-t}^{\lambda} \tau_b(t)dt$. Therefore the total number of cells at time t with generation time $< \lambda$ and age $< a$ equals:

$$\underbrace{n_0 \int_0^{\lambda} \int_0^{a} g(t,x)dtdx}_{\text{Number of cells at } t=0 \text{ with age} < a \text{ and } T < \lambda.} = \underbrace{\int_{-a}^{0} 2k \cdot n_0 \cdot e^{kt}}_{\text{Number of cells with age} < a, \text{ i.e. born after time } t=-a.} \underbrace{\int_{-t}^{\lambda} \tau_b(x)dx}_{\text{Fraction of cells with } T < \lambda.} dt$$
$$= 2n_0 \int_0^{\lambda} \tau_b(x) \left(e^{kx} - e^{-ak}\right) dx. \tag{S13}$$

Differentiation yields:

$$g(a,x) = 2k\tau_b(\lambda)e^{-ak} = k\tau_m(\lambda)e^{k(\lambda-a)}. \tag{S14}$$

8.4 Illustrations of StochPy simulations with cell growth and division

A typical StochPy modeling session consists of first creating a StochPy cell division model object from a (default) input model. Of course, different user-defined models (in SBML or PySCeS MDL) can be loaded into the model object. Once a model is loaded various simulation parameters that are specific for the SSA with cell growth and division can be set, e.g. μ, Ψ_D, and ρ (see also Tabel S2). Kinetic parameter values and molecule copy numbers can be modified interactively, and simulations can be performed by calling the available analysis methods for model objects. As model objects are fully encapsulated, multiple models can be instantiated from the same (or different) input files at the same time. An example of a short StochPy modeling session within Python is:

```python
import stochpy
cmod = stochpy.CellDivision(model_file = "ImmigrationDeath.psc")
cmod.ChangeParameter("Ksyn",2)
cmod.ChangeParameter("Kdeg",0.1)
cmod.SetGrowthFunction(growth_rate=0.1,growth_type="exponential")
cmod.SetVolumeDistributions(phi = ("beta",5,5), K = ("fixed",0.5),
                             phi_beta_mean=2)
cmod.DoCellDivisionStochSim(end=100,mode="generations")
cmod.PlotSpeciesVolumeTimeSeries()
```

where we start by initiating the model object `cmod` for the immigration-death model depicted in PySCeS MDL and modifying the kinetics parameters interactively. Next, we set volume specific growth rate characteristics and volume distributions. Then, we generate one time trajectory with cell growth and division (100 generations) and plot the corresponding (discrete) molecule copy number and volume time series data.

Our lineage corresponds to a sample of mother cells, so we can calculate the statistical properties of e.g. the sample of extant cells via the following high-level functions:

```python
cmod.AnalyzeExtantCells()
cmod.PlotSpeciesExtantDistributions()
```

Here we used the default number of bins for both age and generation times. StochPy generates a warning if the numerical integration was not accurate enough. Choosing a different number of bins for both age and generation time typically solves this issue.

Parameter	Case Study 1	Case Study 2	Case Study 3
k_{syn} (min^{-1})	2.0	2.0	2.0
k_{deg} (min^{-1})	0.1	0	0.1
μ (min^{-1})	0.1	0.1	0.1
V_0 (μm^3)	1.0	1.0	1.0
Ψ_D (μm^3)	Beta(5,5)^i	2	2
ρ	0.5	0.5	0.5

Table S3. Parameters used in the different case studies. We simulated each SSA with cell growth and division for 10^4 generations.

8.5 Comparing StochPy simulations with cell growth and division to analytical solutions

In the following sections, we compare StochPy simulations to theory. In each comparison, we highlight which contributions of cell growth and division to non-genetic variability of cells were included. Besides the simulated results the derived analytical solutions are given. We used the immigration-death model (i.e."synthesis-degradation") model to study single-cell transcription with cell growth and division, because this is the only model for which we know the analytical solutions. We used the settings given in Table S3 to—for molecule copy numbers and cell volume—compare time series, distributions at birth ($a = 0$) and division ($a = T$), and distributions for a sample of extant cells.

8.5.1 Case Study 1: Volume statistics are independent of the model

We first analyzed the volume statistics generated by StochPy simulations. While we used the synthesis-degradation model we could have used any model because the volume statistics are completely independent of the model used. Using a different model and/or parameter settings has only an effect on the run time of the simulation. The settings that we used to generate the results are given in the second column of Table S3.

Comparison of the volume statistics generated by StochPy to analytical solutions shows an excellent overall agreement (Fig. S13). In our stochastic simulation we took into account the following contributions of cell growth and division to non-genetic cellular heterogeneity: imprecise, binomial, partitioning of molecules at cell division and heterogeneity in the mother cell volume at cell division. The latter was modeled by drawing division volumes from a Beta distribution (Eq. (S2)). Imprecise volume division from mother to daughter cells was not taken into account because we partitioned the cellular volume exactly between both daughter cells ($\rho(0.5) = 1$). Heterogeneity in the cell volume at division has several consequences. First, the cellular volume at a given age is variable as is illustrated in Fig. S13A for cells at division ($a = T$) and at birth ($a = 0$). The volume distribution of a sample of extant cells (Fig. S13C) is in agreement with theory. Secondly, the generation time T is not deterministic as is illustrated in Fig. S13B and D. In the latter, we compare the generation time distribution of a sample of extant cells (τ_e) obtained by simulation with theory and found an excellent agreement.

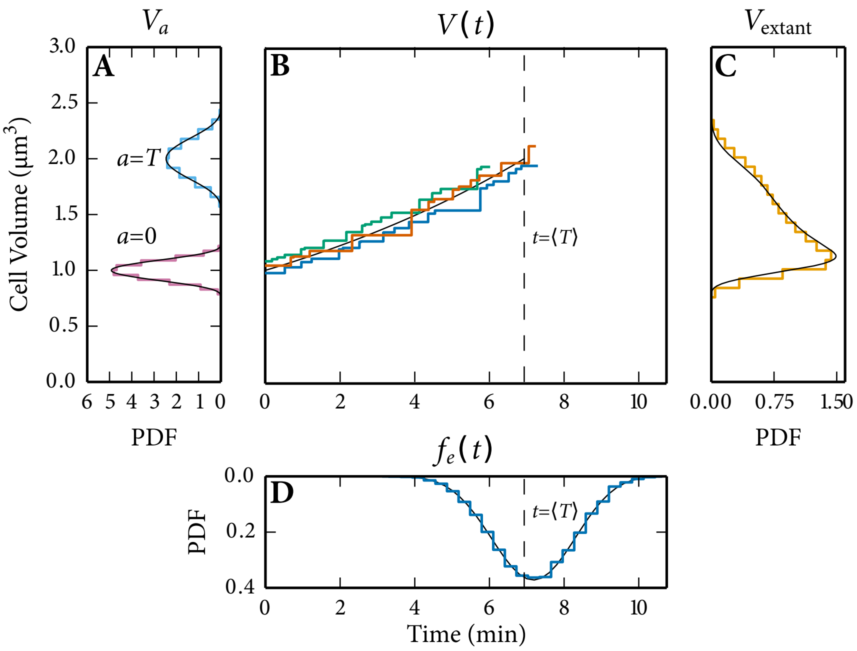

Figure S13. Volume statistics obtained via the SSA with cell growth and division are in agreement with analytical solutions (black). (A) Volume distributions at $a = 0$ and $a = T$. (B) Three stochastic time trajectories of cell volume fluctuate around its analytical solution with $V_0 = 1$. Each of the time trajectories has a generation time that is distributed (see panel D) around its mean, $\langle T \rangle$ (dashed line). (C) The extant volume distribution. (D) The generation time distribution of a sample of extant cells.

In Fig. S13B we simulated three distinct generations that each started with a different V_0 drawn from $\Psi_B(V)$. We selected the parameter values in our model such that the number of firings (reactions that occur) per generation are limited, such that we can illustrate that StochPy updates V only when a reaction fires (Fig. S13B). Our model does not contain any second or higher-order reactions, so this has no effect on the accuracy of the simulation. As explained, this can have an effect if second-order reactions are included and when the rate of firing is slow (then the volume difference becomes significant).

8.5.2 Analytical solutions: Case Study 1 Volume distributions

We drew the cell volume at division (Ψ_D) from a scaled Beta distribution:

$$V_{division} \sim \Psi_D(V) = \frac{(V-1.5)^{\alpha-1}(1-(V-1.5))^{\beta-1}}{B(\alpha,\beta)}, \qquad V \in [1.5, 2.5], \tag{S15}$$

with V as the cell volume and B as the Beta function that normalizes the distribution. Since the beta distribution is defined on the interval [0,1], we scaled this distribution to the interval [1.5,2.5]. When the division volume was reached, we divided the mature mother cell volume equally between both daughter cells (volume partitioning distribution $\rho = 0.5$). The distribution of the volume at birth was obtained by calculating the distribution of the product of two random variables, using Ψ_D and the partitioning distribution ρ. With ρ as the Dirac delta distribution such that $\rho(0.5) = 1$. The cell volume distribution at cell birth is then given by:

$$V_{birth} \sim \Psi_B(V) = 2\Psi_D(2V) = \frac{2^{\alpha}(V-0.75)^{\alpha-1}(2.5-2V)^{\beta-1}}{B(\alpha,\beta)}, \qquad V \in [0.75, 1.25]. \tag{S16}$$

The relationship between the mean of both distributions is given by

$$\langle V_{division} \rangle = \langle V_{birth} \rangle \cdot e^{\mu \langle T \rangle}, \tag{S17}$$

with $T = \ln(2)/\mu$ thus

$$\langle V_{birth} \rangle = \frac{\langle V_{division} \rangle}{e^{\ln 2}} = \frac{1}{2} \langle V_{division} \rangle \,. \tag{S18}$$

The volume distribution of a sample of extant cells can be calculated as function of Ψ_B and Ψ_D following the equations deduced by[29] as shown in[27]:

$$g'(V)\Psi_E(V) + \Psi_E'(V)g(V) = k[2\Psi_B(V) - \Psi_D(V) - \Psi_E(V)] \tag{S19}$$

with $g(x)$ as the growth rate of cells with size V, $\Psi_E(V)$ as volume distribution of a sample of extant cells, Ψ_B as the volume distribution of a sample of baby cells at birth, Ψ_D as the volume distribution at division of a sample of mother cells, and k as the specific growth rate of the population. Assuming $g(V) = k \cdot V$, this function can be simplified to:

$$\Psi_E'(V) = \frac{1}{V}[2\Psi_B(V) - \Psi_D(V) - 2\Psi_E(V)] \tag{S20}$$

which can be solved to give the volume distribution of a sample of extant cells.

Since $V_{division}$ and V_{birth} are independent, the distribution of the ratio $\rho = V_{division}/V_{birth}$ can be calculated from[51]

$$\frac{V_{division}}{V_{birth}} \sim \rho(r) = \int_{-\infty}^{+\infty} |V|\Psi_B(rV) \cdot \Psi_D(V) dV \tag{S21}$$

By using the change of variable technique we can transform $\rho(r)$ and obtain the distribution of the generation time:

$$r = e^{\mu T}, \tag{S22}$$

$$\tau(T) = \left| \frac{\partial}{\partial T} \left(e^{\mu T}\right) \right| \rho(e^{\mu T}). \tag{S23}$$

8.5.3 Case Study 2: mRNA synthesis

In addition to predicting accurate volume statistics, we also aimed at predicting accurate molecule copy number statistics when we include the stochastic contributions of cell growth and division to non-genetic cell variability. Unfortunately, we do not know (yet) the analytical solutions if we include all sources of stochasticity of cell growth and division. Hence, we used a deterministic T and simplified the model by consideration only first-order synthesis of a molecule and no degradation ($K_{deg} = 0$). A deterministic T was achieved by using a fixed Ψ_D and $\rho(r)$ – the specific settings are given in the third column of Table S3. This means that the only contribution of cell growth and division to non-genetic cell variability that we took into account was the imprecise partitioning of molecules at cell division. The analytical solution for this scenario can be found below.

The results given in Fig. S14 show that predictions made with stochastic simulation are consistent with analytical solutions. More specifically, the mRNA copy number distributions at birth and division obtained with stochastic simulations overlap with the theoretical distributions (Fig. S14A) and the time series output of stochastic simulations fluctuate, as expected, around the theoretical time series (Figu. S14B). Here, the deviation is explained by inherent stochasticity in net molecule synthesis in stochastic simulations. We also found an excellent agreement between the simulated and theoretical molecule copy number distribution of a sample of extant cells (Fig. S14C). This result demonstrates that we can simulate a single lineage that represents a sample of mother cells and get information about other samples such as the molecule copy number distribution of a sample of extant cells.

8.5.4 Analytical solutions: Case Study 2 mRNA synthesis

In the second case study, we used a zero-order mRNA synthesis model in exponentially growing cells. With a deterministic generation time, the theoretical mRNA copy number distributions of this model are known[22].

Average molecule copy numbers at birth and division

The mRNA copy number in a cell at a certain age (a) is given by

$$n_a = n_0 + N_a, \tag{S24}$$

where n_a is the mRNA copy number at cell age a, n_0 as the mRNA copy number obtained at cell division, and N_a as the mRNA molecules synthesized since the last cell division at age a. The following relationship holds for the averages

$$\langle n_a \rangle = \langle n_0 \rangle + \langle N_a \rangle. \tag{S25}$$

When symmetric partitioning at the end of a cell cycle ($t = T$) is assumed, the average mRNA copy number at birth is half the average of mRNA copy number at division:

$$\langle n_T \rangle = 2\langle n_0 \rangle. \tag{S26}$$

This means that cells double their average number of molecules during one cell cycle ($a = 0$ to $a = T$). The average amount of molecules produced at $t = T$ equals the average number molecules obtained at $t = 0$.

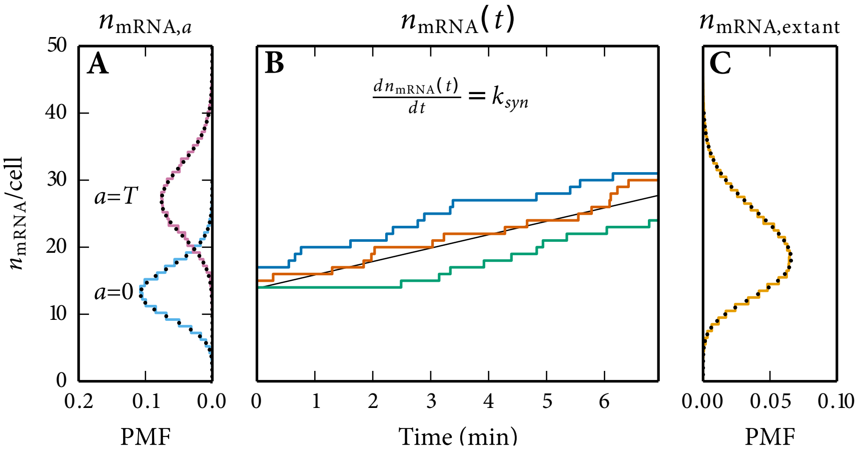

Figure S14. Molecule copy number statistics obtained for the mRNA synthesis model, via the SSA with cell growth and division, is in agreement with analytical solutions. Simulation results are shown in colour, and analytical solutions in black. (A) n_{mRNA} copy number distributions at $a = 0$ and $a = T$. (B) three stochastic time trajectories of n_{mRNA}. (C) the extant n_{mRNA} copy number distribution.

8.5.5 Poisson distributed molecule copy numbers at a specific age

The waiting time distribution of a first-order synthesis process is exponentially distributed, the time between consecutive events is:

$$t_s \sim g(t_s) = k_s \cdot e^{-t_s k_s}. \tag{S27}$$

The time to make N molecules follows a gamma distribution and can be derived from the N-th convolution of $g(t)$ using the generating function, which is the Laplace transform ($\mathscr{L}$):

$$\begin{aligned} G(\chi = N|t) &= \mathscr{L}^{-1}(\mathscr{L}(g(t))^N), \\ &= \frac{e^{-k_s t} k_s^N t^{n-1}}{\Gamma(N)}, \end{aligned} \tag{S28}$$

with $\Gamma[\cdot]$ as the incomplete gamma function. The probability to produce more than N molecules in time t equals:

$$h(\chi \geq N|t) = \int_0^t G(\chi = N|t)dt. \tag{S29}$$

The probability mass function for the production of N molecules at time t is given by:

$$\begin{aligned} h(\chi = N|t) &= h(\chi \geq N|t) - h(\chi \geq N+1|t), \\ &= \frac{e^{-k_s t}(k_s t)^N}{N!}, \end{aligned} \tag{S30}$$

which is a Poisson distribution with mean $k_s t$. Of course, time can be interchanged by age, $t = a$.

8.5.6 mRNA copy number distribution of a sample of extant cells

The molecule copy number distribution of a sample of extant cells was earlier determined by[22]

$$\begin{aligned} p(n) &= \int_0^T u(a) \cdot p(n|a)da, \\ &= \int_0^T u(a) \cdot \frac{e^{-k_s(a+T)}(k_s(a+T))^n}{n!} da, \\ &= \frac{4(k_s T)^n (\Gamma[1+n, k_s T + \ln(2)] - \Gamma[1+n, 2k_s T + \ln(4)]) \ln(2)(k_s T + \ln(2))^{-1-n}}{n!}, \end{aligned} \tag{S31}$$

with $\Gamma[\cdot]$ as the incomplete gamma function, $u(a)$ as the cell age distribution for a population of cells with a deterministic generation time T, and k_s as the synthesis rate constant.

8.5.7 Case Study 3: mRNA synthesis and degradation

Proteins are actively degraded into the cell, so we decided to extent our simple model with active degradation. Except for the degradation rate, we used exactly the same settings (fourth column of Table S3) as in the example without active degradation. The imprecise partitioning of molecules at cell division was, therefore, again the only contribution of cell growth and division to non-genetic variability that was taken into account.

The results given in Fig. S15 show again an almost perfect agreement between stochastic simulation and analytical solutions (species distribution of a sample of extant cells was numerically solved). The corresponding analytical solution can be found below. Comparison of Figs. S14 and S15 shows that, as expected, adding active degradation results in lower mRNA copy numbers at different ages in the lineage and also in the extant cell population.

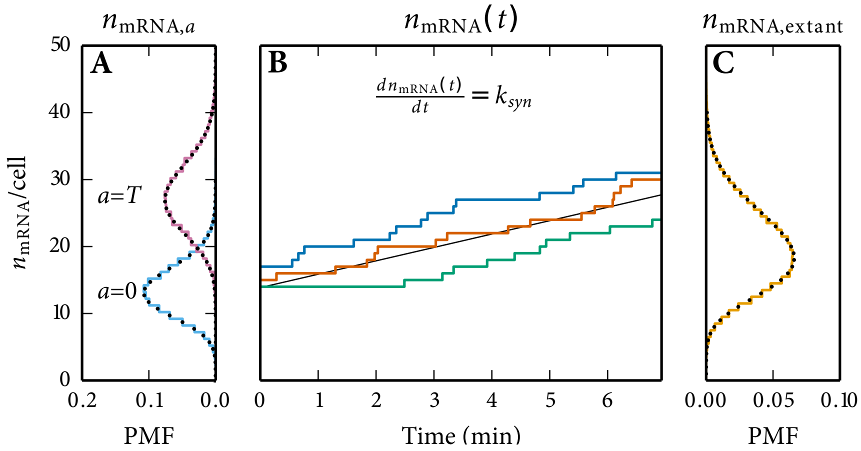

Figure S15. Molecule copy number statistics obtained for the mRNA synthesis and degradation model via the SSA with cell growth and division are in agreement with analytical solutions. Simulation results are shown in colour, and analytical solutions in black. (A) n_{mRNA} copy number distributions at $a = 0$ and $a = T$. (B) three stochastic time trajectories of n_{mRNA} fluctuate around its analytical solution. (C) the extant n_{mRNA} copy number distribution.

8.5.8 Analytical solutions: Case Study 3 mRNA synthesis and degradation

In the third case study, we considered a model consisting of a zero-order mRNA synthesis reaction and first order mRNA degradation reaction, in exponentially growing cells. When we assume a deterministic generation time, the theoretical molecule copy number distributions at a given age of this model are known[22].

8.5.9 Poisson distributed molecule copy numbers at a specific age

With a synthesis and degradation reaction, the average mRNA copy number in a cell of a given age a depends on the mRNA copy number at $t = 0$ and the *net* synthesis until time a. For a linear model with an constant production and degradation rate the average copy number can be written as

$$\langle n_a \rangle = \frac{\int_0^T \left(\langle n_0 \rangle e^{-k_d a} + \frac{k_s}{k_d}(1 - e^{-k_d a}) \right) da}{T}, \tag{S32}$$

where T is the (deterministic) generation time ($T = \ln(2)/\mu$) and $\langle n_0 \rangle$ as:

$$\langle n_0 \rangle = \frac{(e^{k_d T} - 1)k_s}{(2e^{k_s T} - 1)k_d}, \tag{S33}$$

The copy number distribution at age a is given by a Poisson distribution (see[22] for derivation)

$$n_a \sim \text{Poisson}\left[\kappa(a) + \frac{p(a)\frac{1}{2}\kappa(T)}{1 - \frac{1}{2}} p(T) \right] \tag{S34}$$

with the number of molecules produced during a cell cycle as up until age a as $\kappa(a) = \frac{k_s}{k_d}(1 - e^{-k_d a})$ and the survival probability of molecules as $p(a) = e^{k_d a}$.

8.5.10 Molecule copy-number distribution of a sample of extant cells

The molecule copy-number distribution of the sample of extant cells was determined by numerical solving:

$$x \sim \int_0^T p(x|a) \cdot u(a) da, \qquad \text{(S35)}$$

with the $p(x|a)$ determined by Eq. (S34) and the age distribution, $u(a)$, taken from[27] in combination with a deterministic generation time, which then for $u(a)$ gives:

$$u(a) = 2\mu \cdot e^{-\mu \cdot a}, \qquad a \in [0, \frac{ln(2)}{\mu}]. \qquad \text{(S36)}$$
